# Angiogenic potential of skeletal muscle derived extracellular vesicles differs between oxidative and glycolytic muscle tissue in mice

**DOI:** 10.1038/s41598-023-45787-9

**Published:** 2023-11-02

**Authors:** Christopher K. Kargl, Zhihao Jia, Deborah A. Shera, Brian P. Sullivan, Lundon C. Burton, Kun Ho Kim, Yaohui Nie, Monica J. Hubal, Jonathan H. Shannahan, Shihuan Kuang, Timothy P. Gavin

**Affiliations:** 1 Department of Health and Kinesiology, Max E. Wastl Human Performance Laboratory, Purdue University, West Lafayette, IN USA; 2 Department of Animal Sciences, Purdue University, West Lafayette, IN USA; 3 School of Health Sciences, Purdue University, West Lafayette, IN USA; 4 Department of Kinesiology, Indiana University Purdue University Indianapolis, Indianapolis, IN USA

**Keywords:** Physiology, Cell biology

## Abstract

Skeletal muscle fibers regulate surrounding endothelial cells (EC) via secretion of numerous angiogenic factors, including extracellular vesicles (SkM-EV). Muscle fibers are broadly classified as oxidative (OXI) or glycolytic (GLY) depending on their metabolic characteristics. OXI fibers secrete more pro-angiogenic factors and have greater capillary densities than GLY fibers. OXI muscle secretes more EV than GLY, however it is unknown whether muscle metabolic characteristics regulate EV contents and signaling potential. EVs were isolated from primarily oxidative or glycolytic muscle tissue from mice. MicroRNA (miR) contents were determined and endothelial cells were treated with OXI- and GLY-EV to investigate angiogenic signaling potential. There were considerable differences in miR contents between OXI- and GLY-EV and pathway analysis identified that OXI-EV miR were predicted to positively regulate multiple endothelial-specific pathways, compared to GLY-EV. OXI-EV improved in vitro angiogenesis, which may have been mediated through nitric oxide synthase (NOS) related pathways, as treatment of endothelial cells with a non-selective NOS inhibitor abolished the angiogenic benefits of OXI-EV. This is the first report to show widespread differences in miR contents between SkM-EV isolated from metabolically different muscle tissue and the first to demonstrate that oxidative muscle tissue secretes EV with greater angiogenic signaling potential than glycolytic muscle tissue.

## Introduction

Skeletal muscle is a metabolically heterogeneous tissue comprised of different fiber phenotypes. Capillary networks surround muscle fibers, transporting oxygen and substrates to fuel metabolism. Muscle fiber capillary density is tightly regulated and largely reflective of muscle fiber size and metabolic capacity^1^. Oxidative muscle fibers generate ATP predominantly through oxidative phosphorylation, and thus are more oxygen-dependent and contain more mitochondria than predominantly glycolytic fibers. Consequently, oxidative fibers are more highly capillarized than glycolytic fibers, to ensure perfusion matches aerobic capacity^2^.

Skeletal muscle regulates its cellular environment through the secretion of various signaling molecules, known as myokines. Basal capillary density and angiogenic responses to exercise are influenced by intramuscular production and secretion of different pro- and anti-angiogenic myokines. Vascular endothelial growth factor (VEGF) is the most potent of these factors in dictating skeletal muscle capillarity and stimulating exercise-induced angiogenesis. Skeletal muscle VEGF production is vital for basal vascularization, is elevated following exercise and stimulates angiogenesis via induction of capillary hyperpermeability^1,3,4^. Many other factors regulate skeletal muscle angiogenesis and support vascular homeostasis, including: angiopoietin 1 (Ang 1), fibroblast growth factors (FGF), hypoxia-inducible factor-1α (HIF-1α), monocyte chemoattractant protein-1 (MCP-1), interleuken-8 (IL-8), and more^5,6^.

Pro-angiogenic factor expression is generally higher in oxidative fibers compared to glycolytic fibers, consistent with oxidative muscle having greater capillarization and exercise-induced angiogenesis^7,8^. Oxidative muscle produces more VEGF than glycolytic muscle at baseline and following acute exercise^9,10^. Many other angiogenic factors are also greater in oxidative muscle fibers including: HIF-1α, multiple FGFs; receptors for VEGF; MCP-1; and Ang 1, 2, and receptors for Ang^11–13^. Nitric oxide (NO) is an important factor for angiogenesis and endothelial homeostasis. Beyond its vasodilatory action, NO is an endothelial survival factor and coordinates with VEGF during angiogenesis^14–16^. Endothelial nitric oxide synthase (eNOS) and NO production are greater in capillary rich oxidative fibers compared to glycolytic fibers^17,18^.

Extracellular vesicles (EVs) are small membrane bound signaling factors that can biologically effect target cells. Skeletal muscle EVs (SkM-EV) may signal systemically at rest and following exercise^19–21^. However, the majority of SkM-EVs appear to remain in skeletal muscle^21–23^, likely regulating cells in the muscle niche. In vitro*,* SkM-EVs stimulate endothelial cell angiogenesis, potentially through the delivery of microRNAs (miR)^24^. In mice, myogenic cell-derived EVs are taken in by skeletal muscle capillary endothelial cells and are associated with altered gene expression patterns following muscle overload^25^. Skeletal muscle capillary density is proportional to SkM-EV release in mice^24^ suggesting that EVs may regulate muscle capillary endothelial cells. While EV content and signaling effects are generally reflective of the properties of their origin cell^26^, it is unknown whether the content and angiogenic properties of SkM-EVs are regulated by muscle fiber type. Recently we demonstrated that overexpression of peroxisome proliferator-activated receptor gamma coactivator 1-alpha (PGC-1α) in skeletal muscle myotubes enhances the angiogenic properties of myotube-derived EVs^27^. PGC-1α regulates mitochondrial biogenesis and the oxidative phenotype following exercise training. The purpose of the current study was to determine if SkM-EV contents and signaling potential differ between primarily oxidative and glycolytic muscle. We hypothesized that oxidative compared to glycolytic muscle secretes: (1) EVs with greater pro-angiogenic miR content; and (2) EVs with a greater angiogenic response in vitro.

## Results

### Oxidative muscle tissue secretes more EVs than glycolytic muscle tissue

**Figure 1:**
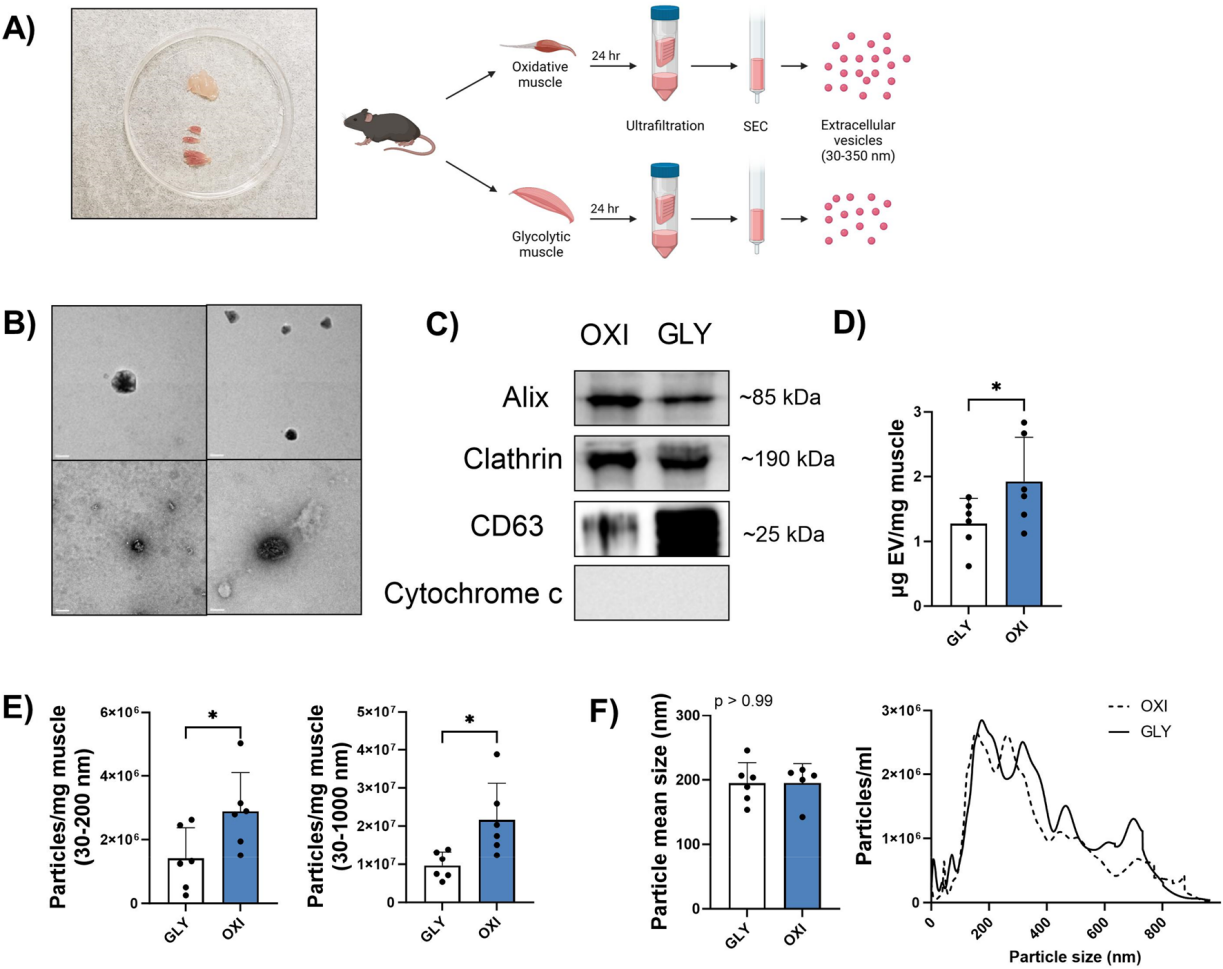
Oxidative muscle secretes more EVs than glycolytic. Representative image of oxidative (OXI) andglycolytic (GLY) muscle groups and schematic of EV isolation protocol (**A**). Transmission electron microscope (TEM) images of OXI- (top panels) and GLY- (bottom) (**B**). Representative western blotting images of EV markers ALIX, clathrin, and CD63 and the negative control cytochrome c; blot images have been cropped to display the bands of interest, full blot images are displayed in supplementary materials (**C**). EV release as measured by total protein concentration, normalized to muscle mass (**D**). EV concentration as measured by nanosight particle analysis at 30–200 nm and 30–1000 nm size ranges (**E**). Mean particle size and representative size distribution graph as measured by nanosight analysis (**F**). Statistical results are the output of paired Student’s t-tests. Mean + SD. *n* = 6 mice/group. Scale bar, 50 nm.

Extracellular vesicles were isolated from primarily oxidative and primarily glycolytic skeletal muscle tissue from mice. A representative image of the isolated muscle and a schematic of the isolation process are in Fig. 1A. Electron microscopy confirmed the presence of small (< 200 nm), circular EVs isolated from oxidative and glycolytic muscle (Fig. 1B; OXI- top images; GLY- bottom images). Additionally, OXI- and GLY-EVs contained robust levels of the EV markers ALIX, clathrin and CD63 and contained no trace of a negative control, cytochrome c (Fig. 1C). When normalized to muscle mass, oxidative muscle secreted 51% more EVs than glycolytic muscle as measured by total EV protein (Fig. 1D). In addition, Nanosight analysis revealed oxidative muscle secreted 106% and 94% more EV sized particles than glycolytic muscle per mass in the 30–200 and 30–1000 nm size ranges, respectively (Fig. 1E). There were no differences in particle mean sizes between the two groups (Fig. 1F).

### miR content differs between oxidative and glycolytic muscle-derived EVs

Small RNA sequencing was performed on isolated EV RNA to determine if EV contents differed by muscle type. Analysis of OXI- and GLY-EV miR sequencing data identified that both groups contained at least a single read of 1,757 different miRs and 297 miRs were significantly different between OXI- and GLY-EVs. Additionally, GLY-EV RNA contained 92% more miR reads than OXI-EVs, despite no difference in total number of RNA reads between the groups (supporting file 1). The sequencing dataset is publicly available through the NCBI GEO database (Accession #: GSE216121). The top 30 differentially expressed miRs (Table 1) were run through Ingenuity Pathway Analysis (IPA). Using a conservative prediction filter (only miRs with experimentally confirmed or highly predictive targets), 3215 mRNAs were identified as targets. Using a p-value filter of p < 0.001, 148 targeted canonical pathways were identified in the dataset. The top 10 canonical pathways are in Table 2. Among the top 10 pathways, four were related to cancer (Glioblastoma, molecular mechanisms of cancer, Chronic Myeloid Leukemia, Pancreatic Adenocarcinoma), two to hepatic signaling (Hepatic Fibrosis, Hepatic Stellate Cell Activation), and four related to growth and cell cycle regulation (Senescence, PTEN, NGF, Cardiac Hypertrophy Signaling).

**Table 1:** Top 30 differentially expressed skeletal muscle extracellular vesicle miRs in oxidative and glycolytic muscle tissue.

Mature miR	Avg. raw counts		log2 fold change	p-value
	OXI-EV	GLY-EV		
mmu-196a-5p	707	6171	− 3.174	2.05E − 15
mmu-100-5p	100	928	− 3.251	6.79E − 15
mmu-26a-5p	1104	9280	− 3.055	2.38E − 13
mmu-148a-3p	109	829	− 2.962	4.068E − 13
mmu-486b-5p	1047	8095	− 2.970	8.183E − 12
mmu-486a-5p	248	2052	− 3.137	1.80E − 11
mmu-199a/199b-3p	743	4238	− 2.544	6.61E − 11
mmu-16-5p	1531	9355	− 2.712	1.492E − 10
mmu-127-3p	123	825	− 2.742	1.72E − 10
mmu-let-7f.-5p	2351	12,620	− 2.424	2.675E − 10
mmu-133a-3p	2316	24,425	− 3.279	3.88E − 10
mmu-181a-5p	102	647	− 2.710	6.95E − 10
mmu-101b-3p	182	1126	− 2.721	9.92E − 10
mmu-99a-5p	125	762	− 2.629	9.11E − 10
mmu-181b-5p	88	560	− 2.755	1.04E − 09
mmu-101a-3p	214	1253	− 2.618	1.126E − 09
mmu-196b-5p	84	500	− 2.641	1.51E − 09
mmu-143-3p	1585	8395	− 2.425	2.57E − 09
mmu-133b-3p	262	2197	− 2.994	4.06E − 09
mmu-125b-5p	1599	8755	− 2.419	6.58E − 09
mmu-26b-5p	329	2104	− 2.648	8.22E − 09
mmu-1a-3p	98,337	429,303	− 2.115	9.44E − 09
mmu-411-5p	20	130	− 2.737	2.51E − 08
mmu-101c	49	269	− 2.480	2.873E − 08
mmu-27b-3p	305	1418	− 2.192	3.62E − 08
mmu-434-3p	124	677	− 2.462	7.63E − 08
mmu-206-3p	194,581	44,127	2.090	1.24E − 07
mmu-29a-3p	652	2932	− 2.256	1.34E − 07
mmu-125a-5p	28	154	− 2.447	1.66E − 07
mmu-181-3p	828	3545	− 2.106	1.85E − 07

**Table 2:** Top 10 significant canonical pathways by p-value from Biological Pathway Analysis of the top 30 differentially expressed skeletal muscle extracellular vesicle miRs in OXI and GLY-EVs.

Canonical pathways	− log(p-value)	Ratio	Z-score (GLY-EV vs OXI-EV)	Molecules
Hepatic fibrosis/hepatic stellate cell activation	14.5	0.356		ACTA2, BAX, BCL2, CCL2, CCN2, CCR5, CCR7, CD40, COL10A1, COL11A1, COL15A1, COL19A1, COL1A1, COL1A2, COL21A1, COL22A1, COL25A1, COL27A1, COL2A1, COL3A1, COL4A1, COL4A2, COL4A4, COL4A5, COL5A1, COL5A2, COL5A3, COL6A3, COL7A1, COL8A1, COL9A1, COL9A2, EDN1, EDNRB, EGFR, FAS, FASLG, FGF1, FGF2, FGFR1, FLT1, IFNG, IGF1, IGF1R, IGFBP3, IGFBP5, IL10, IL1RL1, IL6R, KLF6, MET, MMP13, PDGFA, PDGFB, PDGFC, REL, SMAD3, SMAD4, SMAD7, TGFA, TGFB3, TGFBR1, TGFBR2, TLR4, TNFRSF11B, TNFRSF1A, VCAM1, VEGFA, VEGFC
Senescence pathway	13.7	0.303	− 4.778	ACVR1C, ACVR2A, ACVR2B, AKT3, ANAPC1, ASXL2, ASXL3, BHLHE40, CACNA2D1, CACNA2D3, CACNB2, CAPN7, CBX7, CCND1, CCND2, CCND3, CCNE1, CDC25A, CDC27, CDK6, CDKN1A, CDKN1B, CDKN2A, CDKN2B, CHEK1, DMTF1, E2F3, E2F5, E2F6, E2F7, EED, EIF4E, ELF3, ELF5, ETS1, EZH2, FOXO3, GADD45A, GADD45G, HBP1, IFNA14, IKBKB, ITSN2, JUN, KRAS, MAP2K1, MAP2K4, MAP2K7, MAPK12, MAPK3, MAPK4, MAPK6, MAPK7, MDM2, MRAS, MTOR, NFATC2, NFATC4, NRAS, PDK4, PHF19, PIK3R1, PIK3R2, PPP2CA, PPP2CB, PPP2R5C, PPP3CA, PRKCZ, PTEN, RAF1, RAP1B, RASD2, RB1, RPS6KA5, SAA4, SMAD1, SMAD3, SMAD4, SMAD5, SMAD7, SMAD9, SQSTM1, TGFB3, TGFBR1, TGFBR2, TGFBR3, TP53, VHL, YPEL3, ZFP36L1
Glioblastoma multiforme signaling	12.9	0.357	− 4.901	AKT3, CCND1, CCND2, CCND3, CCNE1, CDC42, CDK6, CDKN1A, CDKN1B, CDKN2A, E2F3, E2F5, E2F6, E2F7, EGFR, FOXO1, FZD3, FZD4, FZD6, FZD8, GRB2, IGF1, IGF1R, KRAS, MAP2K1, MAPK3, MDM2, MRAS, MTOR, MYC, NF2, NRAS, PDGFA, PDGFB, PDGFC, PDIA3, PIK3R1, PIK3R2, PLCL2, PRKCD, PTEN, RAF1, RAP1B, RASD2, RB1, RHOA, RHOB, RHOG, RHOQ, RHOT1, RHOU, SMO, SOS1, SOS2, TP53, TSC1, WNT1, WNT2B, WNT3A, WNT7A, WNT9A
PTEN signaling	11.7	0.36	4.323	BCL2, BCL2L1, CASP3, CCND1, CDKN1A, CDKN1B, CSNK2A1, CSNK2A2, EGFR, FASLG, FLT4, FOXG1, FOXO4, GRB2, GSK3A, IGF1R, IKBKE, INPP5B, INPP5D, INPP5F, INPP5J, ITGA2, KRAS, MRAS, NRAS, PIK3CB, RALA, RALB, RAP1A, RAP1B, RASD2, RELA, RPS6KB1, SIRT6, TGFBR1, TGFBR2, TGFBR3
Hepatic fibrosis signaling pathway	11.6	0.26	− 7.961	ACTA2, ACVR1C, ACVR2A, ACVR2B, AKT3, BCL2, CACNA2D1, CACNA2D3, CACNB2, CASP3, CCL2, CCL3, CCN2, CCND1, CD40, CDC42, CDKN1B, CNR1, COL10A1, COL1A1, COL1A2, COL2A1, COL3A1, COL5A3, CSNK1D, EDN1, EZH2, FGF2, FGFR1, FLT1, FOS, FOXO1, FTL, FZD3, FZD4, FZD6, FZD8, GNAI3, IKBKB, IL18, IL1RL1, IL1RN, IL36G, ITGA2, ITGA6, ITGA9, ITGB3, ITGB4, JUN, KRAS, LRP1, MAP2K1, MAP2K4, MAP2K7, MAPK12, MAPK3, MMP13, MRAS, MTOR, MYC, MYD88, MYLK, NRAS, PDCD4, PDGFA, PDGFB, PDGFC, PIK3R1, PIK3R2, PPARG, PRKAR2A, PRKCD, PRKCZ, PTEN, RAF1, RAP1B, RASD2, REL, RHOA, RHOB, RHOG, RHOQ, RHOT1, RHOU, RIPK1, ROCK1, SMAD3, SMAD4, SMAD7, SMO, SOS1, SOS2, SP1, TFRC, TGFB3, TGFBR1, TGFBR2, TGFBR3, TLR4, TNFRSF11B, TNFRSF1A, VCAM1, VEGFA, VEGFC, WNT1, WNT2B, WNT3A, WNT7A, WNT9A
Molecular mechanisms of cancer	11.2	0.254		APAF1, ARHGEF18, ARHGEF3, ATR, BAK1, BCL2, BCL2L1, BMP1, CAMK2A, CASP3, CCND1, CCND2, CCNE2, CDC25A, CDK11B, CDK14, CDK15, CDK18, CDK19, CDK6, CDK8, CDK9, CDKN1A, CDKN1B, CDKN2B, CRK, CTNNB1, CTNND1, CYCS, E2F5, E2F6, E2F7, ELK1, FADD, FANCD2, FAS, FASLG, FOS, FZD3, FZD6, FZD7, GNA13, GNAI2, GNAT1, GRB2, GSK3A, HIF1A, ITGA2, JUN, KRAS, LRP1, MAPK13, MRAS, MYC, NAIP, NFKBIA, NRAS, PAK1, PIK3CB, PIK3R6, PMAIP1, PRKACA, PRKACB, PRKAR2A, PRKCA, PRKCG, PRKCI, PSENEN, RALA, RALB, RAP1A, RAP1B, RASA1, RASD2, RASGRF1, RELA, RHOA, RHOB, RHOG, RHOJ, RND2, SMAD1, SMAD2, SMAD9, SUV39H1, TAB1, TAB2, TCF4, TGFBR1, TGFBR2, TP53, TYK2, WNT1, WNT11, WNT4, WNT5A, WNT7B, WNT8A, WNT9A
Chronic myeloid leukemia signaling	10.7	0.393		AKT3, BAD, BCL2L1, CCND1, CCND2, CCND3, CDK6, CDKN1A, CDKN1B, CDKN2A, CRK, CRKL, E2F3, E2F5, E2F6, E2F7, GRB2, HDAC4, IKBKB, KRAS, MAP2K1, MAPK3, MDM2, MRAS, MYC, NRAS, PIK3R1, PIK3R2, RAF1, RAP1B, RASD2, RB1, REL, SMAD3, SMAD4, SOS1, SOS2, SUV39H1, TGFB3, TGFBR1, TGFBR2, TP53
Pancreatic adenocarcinoma signaling	10.3	0.365	− 4.49	AKT3, BAD, BCL2, BCL2L1, CCND1, CCNE1, CDC42, CDKN1A, CDKN1B, CDKN2A, CDKN2B, E2F3, E2F5, E2F6, E2F7, EGFR, ERBB2, GRB2, HBEGF, HDAC4, HMOX1, KRAS, MAP2K1, MAP2K4, MAPK12, MAPK3, MDM2, NOTCH1, PDGFC, PIK3R1, PIK3R2, PLD1, PTGS2, RAF1, RB1, REL, SMAD3, SMAD4, SUV39H1, TGFA, TGFB3, TGFBR1, TGFBR2, TP53, VEGFA, VEGFC
NGF signaling	10.3	0.373	− 6.325	AKT3, BAX, CDC42, CRK, GAB1, GRB2, IKBKB, KRAS, MAP2K1, MAP2K4, MAP3K1, MAP3K10, MAP3K11, MAP3K13, MAP3K2, MAP3K5, MAP3K9, MAPK12, MAPK3, MAPK7, MRAS, NGF, NRAS, PDPK1, PIK3R1, PIK3R2, PRKCD, PRKCZ, RAF1, RAP1B, RASD2, REL, RHOA, RHOG, ROCK1, RPS6KA1, RPS6KA3, RPS6KA5, SMPD1, SMPD3, SOS1, SOS2, TP53, TRAF4
Cardiac hypertrophy signaling (Enhanced)	9.6	0.232	− 9.806	ACE, ACVR1, ACVR1C, ADRA2A, ADRB2, AGTR1, APEX1, ATF2, ATP2A2, BORCS8-MEF2B, CAMK2A, CD40LG, CHP1, CTNNB1, DIAPH2, EDN1, EDNRA, EDNRB, EIF2B3, EIF4E, ELK1, ENPP6, FASLG, FGF1, FGF10, FGF16, FGF17, FGF18, FGF20, FGF7, FZD3, FZD6, FZD7, GDPD1, GNA13, GNAI2, GNG2, GNG5, GSK3A, HAND1, HAND2, HDAC4, HDAC7, HSPB2, HSPB7, IGF1, IGF1R, IKBKE, IL13, IL13RA1, IL17C, IL17RD, IL2RB, IL3, IL36G, IL4R, INPP5F, ITGA2, JUN, KRAS, LIF, MAP3K1, MAP3K13, MAP3K2, MAP3K8, MAPK13, MEF2A, MEF2B, MKNK2, MRAS, MYC, NFATC2, NRAS, PDE12, PDE3A, PDE4A, PDE6B, PDE6D, PDE6G, PDE7A, PIK3CB, PIK3R6, PLCD3, PLCH2, PPP3CA, PPP3R1, PPP3R2, PRKACA, PRKACB, PRKAR2A, PRKCA, PRKCG, PRKCI, PTGS2, RALA, RALB, RAP1A, RAP1B, RASD2, RELA, RHOA, ROCK1, RPS6KB1, TGFBR1, TGFBR2, TGFBR3, TNFSF10, TNFSF11, TNFSF13B, TNFSF15, TNFSF9, WNT1, WNT11, WNT4, WNT5A, WNT7B, WNT8A, WNT9A

Ratio = number of molecules in dataset/total number of molecules in the pathway. Z-Score—indicates predicted upregulation or downregulation of pathway glycolytic compared to oxidative muscle EV miR.

**Figure 2:**
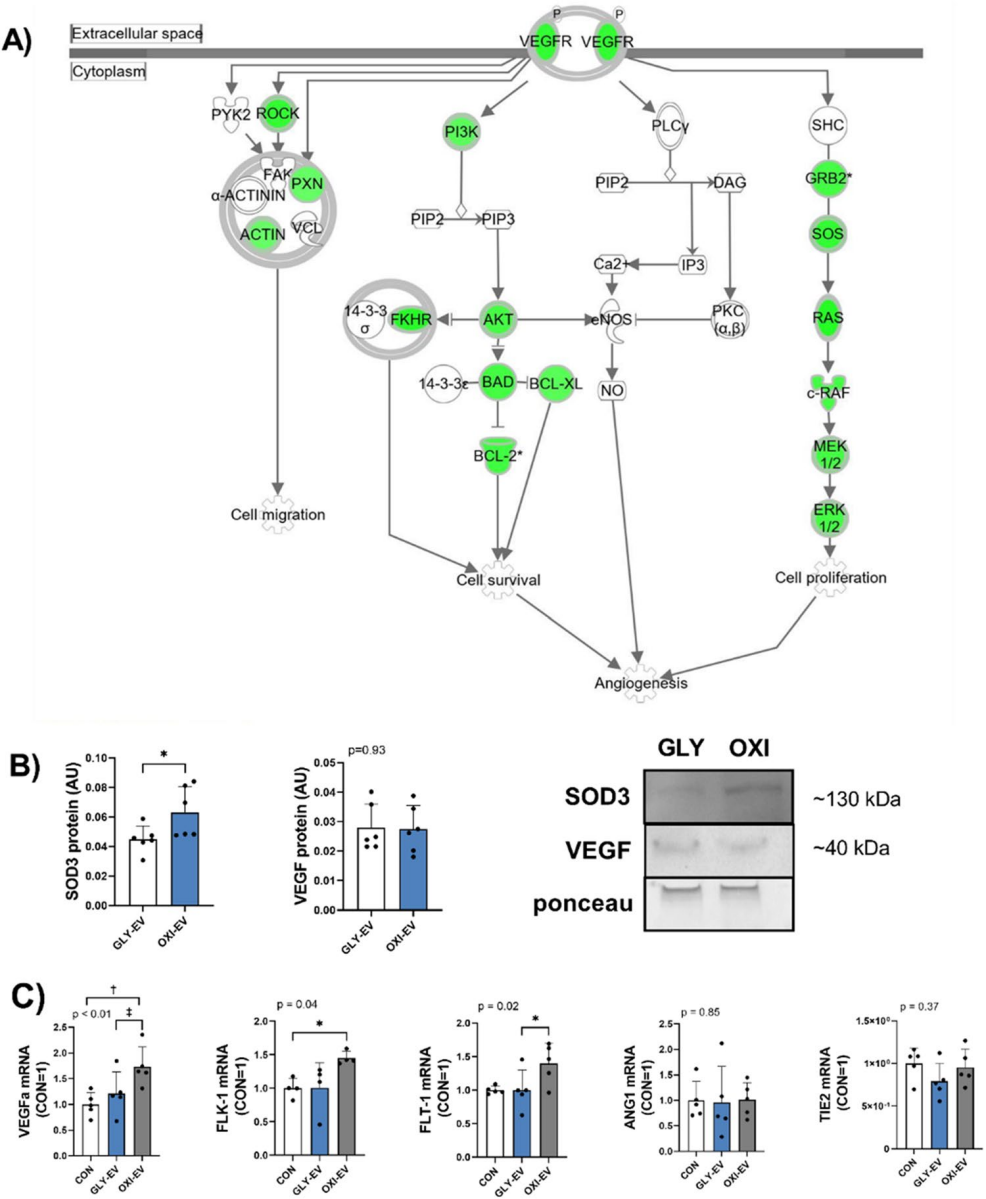
OXI- and GLY-EV miR differentially regulate angiogenic pathways. Ingenuity pathway analysis (IPA) generated vascular endothelial growth factor (VEGF) and angiogenesis pathway (**A**). Green denotes predicted gene downregulation by GLY-EV miR, compared to OXI-EV miR. EV protein contents of VEGF and superoxide dismutase 3 (SOD3), with representative blots, blot images have been cropped to display the bands of interest, full blot images are displayed in supplementary materials (**B**). HUVEC gene expression of angiogenesis-related factors (**C**). Gene expression statistical results are the output of repeated measures one-way ANOVA and are reported as a fold change, untreated control = 1. Mean + SD. *< 0.05; †< 0.01; ‡< 0.001. *n* = 6.

Additionally, several endothelial-relevant signaling pathways were identified among the significant canonical pathways (Table 3). Among these pathways, OXI-EVs were predicted to be less inhibitory to angiogenic signaling and endothelial function than GLY-EVs. VEGFa was a common component of the top endothelial pathways. A representative VEGF angiogenic pathway generated using IPA is depicted in Fig. 2A. Since VEGF and ROS pathways were identified by IPA as targeted pathways, EV contents of superoxide dismutase 3 (SOD3) and VEGF were analyzed. OXI-EV contained more SOD3 protein than GLY-EV (Fig. 2B). There was no difference in VEGF protein content between OXI and GLY-EV (Fig. 2B). Consistent with the IPA prediction, VEGFa and VEGFR1 (FLT-1) mRNA were higher in HUVEC treated with OXI-EV compared to GLY-EV (Fig. 2C). OXI-EV elevated VEGFa and VEGFR2 (FLK-1) gene expression compared to untreated controls. There was no difference in expression of FLK-1 or VE-Cadherin between OXI and GLY-EV. There were no differences in expression of VEGFa, FLT-1, or FLK-1 between GLY-EV and controls. There was no difference in HUVEC gene expression of Ang-1 and Tie-2 between the three groups (Fig. 2C). VE-Cadherin expression was lower in both EV groups compared to untreated controls (Fig. 2C).

**Table 3:** Endothelial specific canonical pathways by p-value from Biological Pathway Analysis of top 30 differentially expressed skeletal muscle extracellular vesicle miRs in OXI and GLY-EVs.

Canonical pathways	− log(p-value)	Ratio	Z-score (GLY-EV vs OXI-EV)	Molecules
HIF1α signaling	4.66	0.24	− 5.94	ADM, AKT3, CDKN1A, EDN1, EGLN2, EIF4E, EPO, FGF2, FLT1, HK2, HMOX1, IGF1, IL6R, JUN, KRAS, MAP2K1, MAP2K4, MAP2K7, MAPK3, MDM2, MET, MKNK1, MKNK2, MMP10, MMP13, MMP14, MMP15, MRAS, MTOR, NRAS, PDGFB, PDGFC, PIK3R1, PIK3R2, PKM, PPP3CA, PRKCD, PRKCZ, RAF1, RAP1B, RASD2, SLC2A1, SLC2A14, SLC2A3, TGFA, TP53, VEGFA, VEGFC, VHL, VIM
VEGF family ligand-receptor interactions	4.16	0.298	− 4.69	AKT3, FLT1, FOS, GRB2, KRAS, MAP2K1, MAPK3, MRAS, NRAS, NRP1, NRP2, PIK3R1, PIK3R2, PLA2G1B, PLA2G2F, PLA2G3, PRKCD, PRKCZ, RAF1, RAP1B, RASD2, SOS1, SOS2, VEGFA, VEGFC
VEGF signaling	4.15	0.283	− 4.379	ACTA2, AKT3, BAD, BCL2, BCL2L1, EIF1AX, ELAVL1, FLT1, FOXO1, FOXO3, GRB2, KRAS, MAP2K1, MAPK3, MRAS, NRAS, PDGFC, PIK3R1, PIK3R2, PXN, RAF1, RAP1B, RASD2, ROCK1, SOS1, SOS2, VEGFA, VEGFC
Production of nitric oxide and reactive oxygen species	4.04	0.236	− 4.111	AKT3, APOC4, ARG2, CDC42, ELP1, FOS, IFNG, IKBKB, JUN, MAP2K1, MAP2K4, MAP2K7, MAP3K1, MAP3K10, MAP3K11, MAP3K13, MAP3K2, MAP3K5, MAP3K9, MAPK12, MAPK3, PIK3R1, PIK3R2, PPP1R10, PPP1R3D, PPP1R7, PPP2CA, PPP2CB, PPP2R5C, PRKCD, PRKCZ, RAP1B, REL, RHOA, RHOB, RHOG, RHOQ, RHOT1, RHOU, S100A8, SAA4, SPI1, TLR4, TNFRSF11B, TNFRSF1A
Apelin endothelial signaling pathway	3.2	0.237	− 4.811	AKT3, APLN, CCL2, FOS, GNAI3, GNG12, GNG13, GNG5, HDAC4, JUN, KLF2, KRAS, MAP2K1, MAP2K4, MAPK12, MAPK3, MEF2A, MEF2B, MEF2C, MRAS, MTOR, NRAS, PIK3R1, PIK3R2, PRKCD, PRKCZ, RAF1, RAP1B, RASD2, REL, SMAD3, SP1, VCAM1

Ratio = number of molecules in dataset/total number of molecules in the pathway. Z-Score – indicates predicted upregulation or downregulation of pathway of glycolytic compared to oxidative muscle EV miR.

### Angiogenic properties of OXI- and GLY-EV

**Figure 3:**
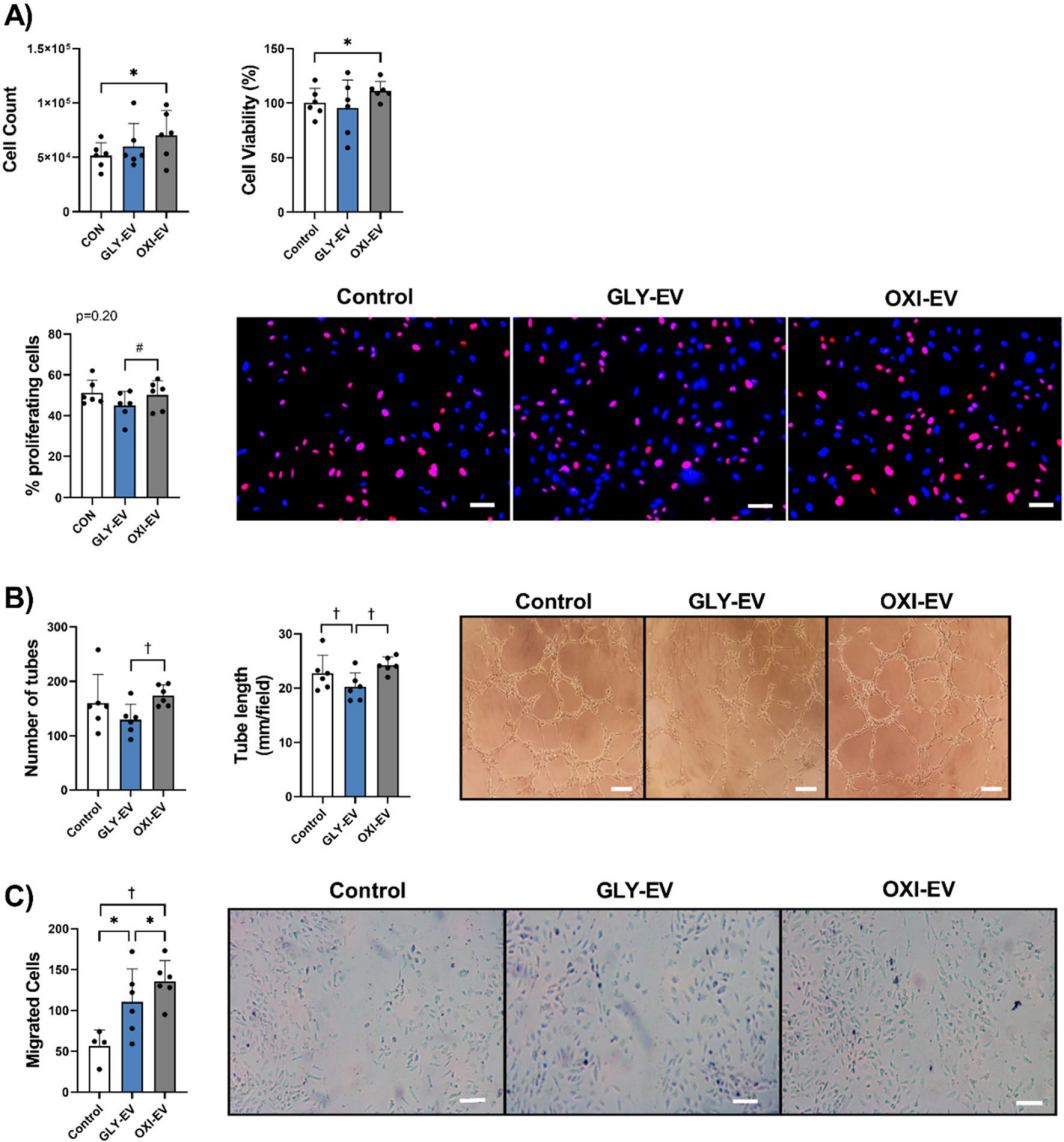
OXI-EV enhances EC migration and tube formation. Human umbilical vein endothelial cell (HUVEC) count, viability, and proliferation (% EdU + nuclei/total nuclei) following 48 h EV treatment (**A**). HUVEC tube formation with representative images (**B**). HUVEC migration with representative images (**C**). statistical results are the output of repeated measures one-way ANOVA with Tukey’s posthoc. Control represents HUVECs treated with vehicle only, no EVs. Scale bars = 200 μm. Mean + SD. *< 0.05; †< 0.01; *n* = 6.

Cultured endothelial cells were treated with OXI and GLY-EV to determine if they altered EC angiogenic properties in vitro*.* OXI-EV, but not GLY-EV increased HUVEC number and cell viability compared to controls after a 48 h incubation (Fig. 3A). There was no difference in HUVEC number or viability between OXI-EV and GLY-EV. There was no difference in cell proliferation between the groups (Fig. 3A). HUVECs treated with OXI-EVs formed more tubules and had a greater tube length than GLY-EV treated HUVECs. Tube formation was not different between OXI-EV and untreated HUVECs (Fig. 3B). HUVEC treated with OXI-EV demonstrated greater tube formation and tube length than HUVEC treated with GLY-EV (Fig. 3B). On the endothelial cell scratch assay, OXI-EVs promoted greater HUVEC migration than GLY-EVs; both EV conditions elevated HUVEC migration compared to untreated controls (Fig. 3C).

### Nitric oxide pathways mediate pro-angiogenic properties of OXI-EV

**Figure 4:**
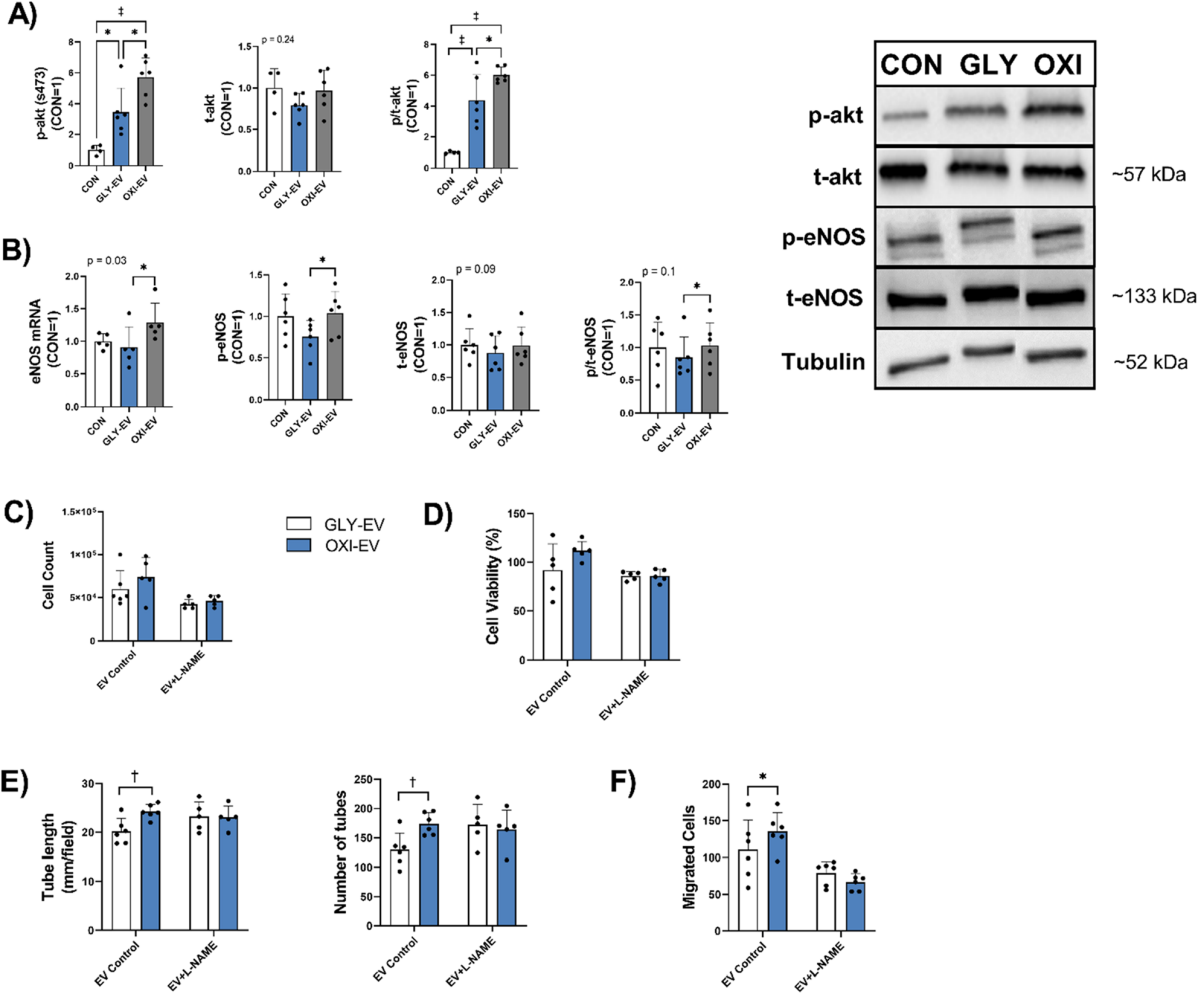
OXI-EV induced angiogenesis is mediated via NO signaling pathways. Protein expression of total and phosphorylated Akt, with representative blots (**A**). Gene and protein expression of total and phosphorylated endothelial nitric oxide synthase, with representative blots (**B**). Blot images have been cropped and arranged so that all bands of interest are displayed in the same order, full blot images are displayed in supplementary materials. HUVEC counts (**C**), viability (**D**), tube length and number of tubes (**E**), and migrated cells (**F**) under control and L-NAME conditions in the presence of EV treatments. Gene expression is reported as a fold change, with untreated controls = 1. Statistical results are the output of repeated measures one-way ANOVAs and student’s t-tests (EV + L-NAME comparisons). Mean + SD. *< 0.05; †< 0.01; ‡< 0.001. *n* = 6.

The nitric oxide (NO) pathway was one of the endothelial-relevant pathways predicted by IPA to be positively regulated by OXI-EV compared to GLY-EV (Table 3). This pathway is mediated by eNOS and is important in the regulation of endothelial cell function and angiogenesis. The Akt/eNOS pathway was evaluated to determine whether OXI and GLY-EV differentially regulated the key intracellular endothelial NOS pathway. Akt phosphorylation at serine 473 was significantly greater in OXI-EV compared to GLY-EV treated ECs, both EV conditions increased p-Akt compared to controls (Fig. 4A). There was no difference in total amounts of Akt across the groups (Fig. 4a). eNOS mRNA was lower in HUVEC treated with GLY-EV compared to OXI-EV; with no other differences between groups (Fig. 4B). Additionally, eNOS phosphorylation and eNOS phosphorylation/total ratio were greater in OXI-EV compared to GLY-EV treated ECs; with no other differences between the groups and no differences in total eNOS (Fig. 4B).

To identify the contribution of NOS to angiogenesis in OXI- and GLY-EV treated HUVECs, HUVEC were co-incubated with EV and the NOS antagonist L-NG-Nitro arginine methyl ester (L-NAME) and compared to EV-treated control cells. There was no difference in cell viability and cell counts between GLY- and OXI-EV treated cells following L-NAME treatment (Fig. 4C,D). L-NAME treatment eliminated muscle specific EV differences in HUVEC tube formation/length and number of migrated cells following scratch assay (Fig. 4E,F).

## Discussion

The novel findings of the current study are: (1) OXI-EV promote greater endothelial cell migration and tube formation compared to GLY-EV; (2) miR content differs between OXI-EV and GLY-EV demonstrating heterogeneous EV content based on fiber type; and (3) greater OXI-EV angiogenesis may be mediated through NOS-regulated mechanisms. We also confirm our previous findings that oxidative muscle secretes more EVs than glycolytic muscle tissue^24^. The combination of greater EV secretion along with more pro-angiogenic EV is consistent with greater capillarization of oxidative compared to glycolytic muscle fibers.

### Oxidative muscle tissue releases more EVs than glycolytic muscle tissue

The findings in the current study are consistent with our prior work showing oxidative muscle tissue secretes more EVs than glycolytic muscle tissue^24^. Previously, we measured EV release through acetylcholinesterase activity and EV marker protein content. We have added to this data by measuring EV concentration through nanoparticle tracking analysis and total protein measurements, in line with recommendations from the International Society of Extracellular Vesicles^28^. Isolated EV fractions from both groups had a strong presence of several EV markers, while containing no measurable amount of cytochrome c, as expected. It has been previously reported the soleus secretes nearly twenty times more EVs/mg muscle than the quadriceps^29^. However, in that report muscle was not teased apart during EV collection, limiting the surface area of the tissue and potentially leading to hypoxia-induced EV release with an unknown outcome on muscle specific differences^29^. A hypoxic muscle environment may have been present for a short time in the present study as well, as mice were euthanized via CO_2_, before the muscle was quickly harvested, teased apart and incubated in normoxic conditions. The magnitude of our findings however, are in closer agreement with the soleus secreting more EVs than the glycolytic plantaris as demonstrated by Estrada and colleagues^23^. Consistent evidence strongly suggests that oxidative muscle secretes more EVs than glycolytic muscle.

Metabolic capacity could regulate EV release as highly metabolic skeletal muscle secretes nearly 100 fold more EVs than adipose tissue^23^ and increased metabolism in cancer cells increases EV release^30^. Metabolic activity likely explains the increase in EV release from skeletal muscle and other cells during acute exercise as well^21,31^. Multivesicular body (MVB) generation, a primary mechanism of EV release, is regulated by cellular energy flux. Low energy states and autophagy promote MVB recycling while high energy states promote MVB-plasma membrane docking and EV release^32^. Previously we found that muscle satellite cell senescence elevates EV release^33^. Despite reductions in proliferation, senescence induces high metabolic activity characterized by mitochondrial dysfunction with elevated reactive oxygen species (ROS) and cytokine production^34^. Conversely, skeletal muscle from leptin-deficient obese mice releases fewer EVs than control muscle. Leptin deficiency disrupts glucose and lipid metabolism resulting in a state of reduced energy expenditure and muscle atrophy^35,36^. While greater metabolic capacity of oxidative muscle fibers may explain enhanced EV release compared to glycolytic fibers, it appears not to be directly regulated by PGC-1α as its overexpression does not alter SkM-EV release *in vitro*^27^.

### Oxidative muscle-EVs have greater angiogenic potential than glycolytic muscle EVs

Skeletal muscle EV-specific membrane labeling in mice has shown that SkM-EVs are targeted to capillary ECs following muscle overload. Previously we found that EVs from C2C12 myotubes increase EC growth, migration, and tube formation^24^. Oxidative muscle fibers have a more pro-angiogenic secretory phenotype than glycolytic fibers^9–11,13^ and PGC-1α, a transcriptional regulator of the oxidative phenotype, regulates the angiogenic potential of SkM-EVs^27^. Consequently, we hypothesized that OXI-EV would promote greater in vitro angiogenesis than GLY-EV. In the current report, HUVEC were treated with equal amounts of OXI- and GLY-EVs to determine EV angiogenic potential. OXI-EV increased EC migration, number of tubules, tubule length and trended towards increased proliferation compared to GLY-EVs on a per EV basis. Combined with greater EV release from oxidative muscle, we may be underestimating the pro-angiogenic benefits of OXI-EVs occurring in vivo. Murach et al*.* found EV uptake by muscle endothelial cells in vivo is not uniform, with different cells having either high, low, or no EV uptake^25^. In their report, ECs with high EV uptake had greater activation of angiogenesis promoting pathways following muscle overload than ECs with low or no uptake. Since oxidative muscle fibers secrete more EVs than glycolytic fibers, it is possible that there is a greater percentage of high uptake capillaries in oxidative compared to glycolytic muscle.

Importantly, the EVs from the current study were isolated from resting muscle. Therefore, our data support the hypothesis that EVs from oxidative muscle tissue support maintenance of a denser capillary bed in skeletal muscle. The extent to which exercise regulates skeletal muscle-derived EV contents and angiogenic potential remains poorly studied. For example, acute exercise causes a release of SkM-EVs^21,22^, but it is unclear if this is due to depletion of existing muscle EVs or production of new EVs. It is unknown if EVs in resting muscle contribute to early adaptive phases of exercise. Establishing definitive timelines of how exercise impacts EV signaling locally and systemically will be key to determining how EVs contribute to exercise adaptations.

### miR contents differ between oxidative and glycolytic muscle EVs

miR are abundant in EVs and alter the biological function of recipient cells, including endothelial cells^37^. EVs from oxidative and glycolytic muscle contained at least a single read of 1,757 different miRs and 297 miRs were differentially expressed between OXI- and GLY-EVs. As would be expected, the most abundant miRs in OXI- and GLY-EV were miRs highly expressed in skeletal muscle, known as myomiRs^38^. Although the total number of sequencing reads was not different between groups, GLY-EVs contained nearly double the myomiR reads with more miR-1, -133a, and -133b, but less miR-206 than OXI-EVs. Our myomiR data is consistent with previous PCR analysis of EV isolated from the quadriceps and the soleus^29^. OXI-EV contained over four times the amount of miR-206, which is unsurprising as miR-206 is enriched in oxidative fibers and regulates the oxidative phenotype^39^. Delivery of SkM-EV miR-206 to neighboring muscle fibers may help maintain an oxidative phenotype. Differences in miR content between OXI- and GLY-EVs suggest that SkM-EVs are heterogeneous and may have varied signaling roles.

IPA was utilized to generate pathways predicted to be regulated differently between OXI- and GLY miRs. We focused on EC signaling pathways predicted to be differentially regulated, since EV angiogenic potential was our primary objective. Several endothelial regulating pathways were predicted to be positively regulated by OXI-EVs, compared to GLY-EVs, including HIF-1-α, VEGF signaling, VEGF ligand-receptor interactions, NO and ROS production, and endothelial apelin signaling. These pathways regulate EC proliferation, survival, migration, and tube formation^14,40,41^. Pathway analysis predicted lower VEGF and VEGFR expression in GLY-EV compared to OXI-EV treated ECs. Consistent with this, VEGF and VEGFR1 mRNA were lower in HUVEC following GLY-EV treatment. While many of the miRs in OXI- and GLY-EVs have been shown to regulate angiogenesis, miR-16 was highly enriched in both groups and was 6.5-fold higher in GLY-EVs. miR-16 targets VEGF, and an increase in miR-16 in the soleus of hypertensive rats is associated with nearly 50% reductions in VEGF mRNA and capillary-to-fiber ratio^42^. Exercise training reduces miR-16 expression in the soleus, creating a more angiogenic environment by removing a post-transcriptional repressor of VEGF^42^. High levels of anti-angiogenic miRs in GLY-EVs may act similarly by maintaining a baseline level of post-transcriptional repression that is not observed in highly capillarized oxidative muscle tissues. Although the differences in miR contents between OXI and GLY-EVs are stark, they may not completely explain the effects of OXI and GLY-EVs on endothelial cells. In EV treated endothelial cells VEGF, VEGFR, and eNOS mRNA expression were lower in GLY-EV compared to OXI-EV treated cells, as predicted by IPA. However, there was no difference between GLY-EV and untreated controls cells, suggesting that either EV-derived miRs do not have effects on target cell levels of these mRNAs or there are other non-miR factors in EVs contributing to these effects.

### NOS inhibition ameliorates angiogenic benefits of oxidative muscle EVs

Nitric oxide synthase is important in facilitating muscle-regulated angiogenesis^40,41^, where NO regulates endothelial cell homeostasis and promotes cell growth, migration, and tube formation^14–16^. In endothelial cells, eNOS is phosphorylated at ser1177 by Akt, and increased p-Akt at ser473 leads to greater eNOS phosphorylation^43^. In the current study, HUVECs treated with OXI-EV, compared to GLY-EVs, had greater Akt phosphorylation at ser473. Additionally, GLY- and OXI-EV treated cells had much greater Akt phosphorylation compared to control cells. Akt regulated pathways have frequently been identified to be altered by EVs in other cell types^44–46^. OXI-EV treatment increased eNOS phosphorylation in HUVEC compared to GLY-EV, likely due to increased Akt phosphorylation. Despite greater Akt phosphorylation, there were no differences in p-eNOS between OXI-EV treated ECs and untreated controls, suggesting that the GLY-EVs inhibit eNOS phosphorylation. The mechanism responsible for this inhibitory effect is unknown. Direct inhibition by miRs is unlikely as miRs do not act directly on proteins. Indirect regulation may explain the results as multiple genes upstream of eNOS were predicted targets of GLY-EV miRs and a NOS pathway was predicted to be downregulated in GLY-EV, compared to OXI-EV, treated cells. To determine if NOS was important for EV associated angiogenic differences, we incubated HUVEC with EV and the NOS inhibitor L-NAME. L-NAME treatment eliminated muscle specific EV differences in HUVEC tube formation and migration, indicating that pro-angiogenic benefits of OXI-EVs could be mediated through NOS pathways. Alternatively, the L-NAME treatment in HUVECs did not completely inhibit in vitro measures of angiogenesis compared to untreated cells, suggesting that this pathway may not have been sufficiently suppressed and that the angiogenic benefits of OXI compared to GLY-EVs could be mediated through other pathways. More complete mechanistic work is warranted to determine the precise way in which EVs regulate intracellular mechanisms.

While EV protein content was not the main focus of this report, we did find greater SOD3 protein in OXI compared to GLY-EVs. SOD3 is the extracellular superoxide dismutase and converts superoxide to hydrogen peroxide (H2O2). SOD3 is elevated in circulating EVs following exercise^47^. Additionally, pro-angiogenic benefits of plasma EVs are SOD3 dependent as a result of the signaling effects of H_2_O_2_^47^. Similarly, higher SOD3 levels in oxidative muscle tissue derived-EVs are associated with improved aspects of angiogenesis. SOD3 transport is a potential mechanism through which Akt phosphorylation was elevated by OXI-EVs, as SOD3 has been shown to regulate signal transduction pathways, including Akt^48^. To interrogate Akt/eNOS phosphorylation changes further, we probed for VEGF in EVs as VEGF is a powerful activator of Akt/eNOS signaling in endothelial cells^15,49^. Despite being more highly expressed in oxidative muscle fibers^9,10^, we found no difference in VEGF contents between OXI and GLY-EVs. Previously we found that C2C12 EV-mediated angiogenesis was VEGF independent^24^. Circulating EV stimulate angiogenesis similarly to recombinant VEGF, however EVs elicit distinct changes in EC gene expression^50^. Mesenchymal stem cell EVs are pro-angiogenic, but carry significantly less VEGF than is secreted by origin cells^51^. We hypothesize that fiber-derived VEGF and EV secreted into the muscle interstitial space contribute to a coordinated angiogenic effect. The combination of greater VEGF production^9,10^, greater EV release, and the release of EVs with higher angiogenic potential may help oxidative muscle fibers regulate and maintain a denser microvasculature than glycolytic fibers.

### Predicted pathway regulation differs highly between oxidative and glycolytic muscle EVs

While the primary objective of this report was to examine SkM-EV regulation of ECs, our data may provide insight as to how OXI- and GLY-EVs regulate other cell types. The top-targeted pathways were related to cell proliferation, activation, and cancer signaling. It should be noted that IPA identifies target pathways partially through the proportion of the mRNA constituents of a known pathway that are targeted by the given miRs. Therefore, the presence of cancer signaling pathways does not indicate that SkM-EVs are oncogenic in vivo*.* Most of the canonical pathways predicted to be downregulated by GLY-EVs are involved in growth factor production and proliferation. Regulation of these pathways by OXI-EVs will likely not cause growth in unperturbed adult muscle—as muscle does not hypertrophy without a sufficient stimuli^52^. However, a signaling phenotype that supports growth pathways may help resist atrophic states. Indeed, oxidative muscle fibers are more resistant to disease-related muscle atrophy than glycolytic fibers, likely mediated through PGC-1α signaling^53–55^. Recently we have demonstrated that PGC-1α overexpression in human myotubes enhances the angiogenic potential of myotube-derived EVs and protects cultured endothelial cells against an oxidative stress challenge^27^. Future studies should evaluate whether PGC-1α regulation of EV signaling confers similar benefits to other skeletal muscle cell types.

The systemic signaling impact of OXI- and GLY-EVs is beyond the scope of this report, however skeletal muscle EVs may reach the circulation at low quantities and have systemic signaling effects^21–23^. Skeletal muscle-derived EVs have been found in adipose tissue, cardiac tissue, splenocytes, hepatocytes, and dorsal root ganglia neurons^20,21,23^. The top canonically targeted pathways by EV miR include multiple pathways in these tissues, including cardiac hypertrophy pathway, neural growth factor (NGF) pathway, and multiple hepatic pathways. While our data do not demonstrate that OXI- or GLY-EVs specifically target these cells, it supports the notion that the miR contents of SkM-EVs have the potential to regulate common pathways systemically in tissues known to take in SkM-EVs.

## Limitations

Our data provide evidence that oxidative muscle from mice secretes extracellular vesicles that promote aspects of angiogenesis in cultured endothelial cells. A couple features of the model should be emphasized to put the results in proper context. First, fiber-type differences between muscle groups are more prominent in mouse muscle, compared to human muscle. This could make it more difficult to observe the magnitude of differences in EV contents observed in the current study in human muscle because of the mixed fiber type nature of human muscle. Isolating EVs from single muscle fibers or comparing populations with different percentages of oxidative and glycolytic fibers (i.e. endurance athletes vs. untrained) could be informative ways to determine if skeletal muscle metabolic phenotype impacts EV contents and angiogenic signaling in humans. An additional limitation is that EVs were isolated from whole muscle and not specifically from muscle fibers. Consequently, our EV fraction may have contained a small percentage of vesicles derived from other cell types including fibroblasts, endothelial cells, satellite cells, and macrophages. However, the vast majority of EVs are likely derived from skeletal muscle fibers as muscle fibers are the dominant cell type in muscle tissue and the most enriched miRs in the isolated EVs were muscle-specific. The current report only used male mice and while both sexes display higher capillarization around predominantly oxidative muscle fibers, future reports could consider if sex-based differences exist in this signaling axis. Lastly, EVs were isolated from mouse muscle and used to treat human cells. This cross-species model may have resulted in missed interactions or predicted mRNA targets however these concerns would be expected to be limited as miRs are well conserved.

## Conclusion

Numerous pro-angiogenic factors, including VEGF, are produced more abundantly in oxidative compared to glycolytic fibers, likely contributing to greater capillary density in oxidative muscle^10–13^. Together with our previous findings^24^, we demonstrate that oxidative fiber-derived EVs are secreted more abundantly and are more potent stimulators of endothelial cell tube formation and migration than glycolytic fiber EVs. Additionally, OXI-EVs shuttle miRs that greater support pro-angiogenic pathways compared to GLY-EVs. While this data is descriptive, it underlines a novel phenotypic difference between the two tissue types in regards to EV contents. Persistent secretion of pro-angiogenic EVs likely contribute to the higher capillary density surrounding oxidative compared to glycolytic muscle fibers. We also demonstrate that skeletal muscle EVs are not a homogenous population and future work investigating the mechanisms responsible for the differences between oxidative and glycolytic muscle EV release and content will increase our understanding of the physiological contributions of EV to muscle regulation.

## Methods

### Ethical approval

Animal care and procedures were in accordance with the 'European Convention for the Protection of Vertebrate Animals used for Experimental and other Scientific Purposes' (Council of Europe No 123, Strasbourg 1985) and comply with the ARRIVE guidelines. Animal maintenance and experimental protocols were approved by the Purdue University Animal Care and Use Committee.

### Animals and muscle isolation

Male C57BL/6 J mice (00,664) were purchased from Jackson Laboratory at 10–12 weeks old and housed in the Purdue University Animal facility at a controlled temperature (22 ± 2 °C) with 12 h–12 h light–dark cycles. Mice were fed standard rodent chow with free water access. Mice were euthanized via inhalation of carbon dioxide (CO_2_) for 3 min until mice exhibited lack of respiration and had a faded eye color, followed by cervical dislocation before muscle isolation. Glycolytic and oxidative muscles were separated according to anatomical location and colour. Muscles were collected from a single leg from each animal and not pooled. Oxidative muscle consisted of the entire soleus and the red portions of the gastrocnemius. These muscle groups were combined to improve EV yields, as both have been shown to be highly oxidative and rich in mitochondria. Glycolytic muscle consisted of the whole quadriceps. These muscle groups have been shown to be representative of oxidative and glycolytic phenotypes, respectively^56,57^.

### EV isolation

Isolated muscle was gently teased apart with tweezers to increase surface area and incubated in serum-free DMEM for 24 h. The media was filtered through a 100 μm cell strainer and the EVs were isolated using an ultrafiltration-size exclusion chromatography technique (UF-SEC) as previously described^58^. Briefly, the media was centrifuged at 2000 × *g* for 10 min and pelleted cell debris was discarded. The media was then concentrated to 500 μl using Amicon Ultra-15 centrifugal 50 kDa filter tubes (Millipore Sigma, St. Louis, MO, USA). The concentrated media was run through qEVoriginal 35 nm SEC (Izon Science, Medford, MA, USA), using sterile filtered PBS as an elution buffer. According to the manufacturer’s instructions, a 3 ml void volume was collected and discarded prior to collection of the 1.5 ml EV fraction, containing EVs between 35 and 350 nm in diameter. Following collection of the EV fraction, a 6 ml protein fraction was collected before the column was flushed. The EV fraction was collected and stored at – 80 °C.

### EV visualization

Visualization of EVs was performed on a Tecnai T20 transmission electron microscope (TEM; FEI, 200 kV). Briefly, OXI- and GLY-EVs in PBS were pipetted onto carbon coated, copper electron microscopy grids and incubated for approximately 2 min. Excess liquid was blotted away, and grids washed with water to remove salts. Excess liquid was blotted away, and grids were negatively stained with 2% phosphotungstic acid for 1 min. The presence of OXI- and GLY-EVs were further confirmed through immunoblot analysis of several well-known EV markers (ALIX, CD63, and clathrin) and a negative control marker that is not expressed in EV (cytochrome C). EV samples were pooled for immunoblot characterization to increase yield and ensure bands were visible. Immunoblots were not used as quantitative measures of EV release. Six mice/group were utilized for all EV isolations, treatments and assays with the exception of EV miR sequencing (n = 4).

### EV release

EV release was measured using two methods as recommended by the International Society of Extracellular Vesicles^28^ and as previously performed^33^. First, EVs were diluted in sterile filtered PBS to a final volume of 1000 μl. Particle counting and size characterization was performed using a NanoSight LM10 Nanoparticle Tracking Analysis system (Malvern Panalytical, Westborough, MA, USA) equipped with a 405 nm laser and a sCMOS camera. Following auto-setup, camera level and detection thresholds were set to 10 and all other settings were set to the software defaulted auto settings. EVs were loaded into a syringe, pumped into the chamber, and five 30-s videos were captured for analysis. Particle size distribution and concentration per particle size were analyzed using NTA 2.3 software (Malvern Panalytical). Second, OXI- and GLY-EV release was analyzed via EV protein concentration measurement using a BCA kit (ThermoFisher Scientific, Waltham, MA, USA). For both measurements, EV release was normalized to muscle weight.

### Small RNA isolation and sequencing

Total RNA was isolated from oxidative and glycolytic muscle EVs (n = 4/group) using miRNeasy micro kits (Qiagen, Germantown MD, USA), according to the manufacturer’s instructions. Total RNA was resuspended in UltraPure water (Invitrogen, Waltham, MA, USA). Following quality control testing, EV small RNA was sequenced by the Center for Medical Genomics at Indiana University School of Medicine. Briefly, 3–8 ng of total RNA/sample were sequenced using a NextSeq 500/550 high output v2 kit (Illumina, San Diego, CA, USA). HO 75 cycle flow cells were utilized and sequencing was performed at 75,000,000 reads per cell. Following sequencing, raw RNA reads were analyzed by the Bioinformatics core at Indiana University School of Medicine. GEO accession number for sequencing dataset: GSE216121.

### Biological pathway analysis

Bioinformatics identified 297 differentially expressed (p < 0.05) miRs between OXI- and GLY-EVs. To return a manageable number of target mRNAs for biological pathway analysis, the 30 top differentially expressed miRs were uploaded to Ingenuity Pathway Analysis (IPA; Qiagen). IPA identifies miR:mRNA target interactions across multiple bioinformatics databases. A conservative approach was used for the mRNA target prediction where only highly predictive and/or experimentally observed targets were selected as determined via miRTarBase and TargetScan respectively^59,60^. This approach identified a gene target list of 3215 mRNAs. The targeted mRNA dataset was utilized by IPA to identify canonical pathways predicted to be regulated differently by OXI- and GLY-EV miR. Canonical pathways were tested using Fisher Exact Test of ratios of miR targeted genes in our dataset as compared to the total number of genes in each IPA pathway.

### Cell culture treatments

Human umbilical vein endothelial cells from a single donor were purchased (HUVECs; Cell Applications, San Diego, CA, USA) and amplified in endothelial cell growth media (EGM; Cell Applications) and seeded into 24-well cell culture plates (10,000 cells/well) for all experimental assays and into 6-well plates (50,000 cells/well) for protein and RNA analyses. EV treatments were done in Human Endothelial Cell Basal media (Cell Applications; EBM) supplemented with 2% EV-depleted FBS, unless otherwise noted. OXI- and GLY-EV treatments were done at a concentration of 10 μg/ml in equalized volumes of PBS, as this has previously been an effective minimum EV dose for altering HUVEC function^33^. Control cells were treated with equal volumes of PBS vehicle. EV treatment times were dependent upon the assay, ranging from 6 to 48 h. A subset of cells were treated with the nitric oxide synthase (NOS) antagonist L-NG-Nitro arginine methyl ester (L-NAME). When appropriate, HUVECs were treated with 200μM L-NAME (ThermoFisher Scientific) immediately prior to EV treatments.

### Cell counts and viability

Cell viability was assessed via a 3‐ (4, 5‐dimethylthiazol‐2‐yl) ‐2, 5‐diphenyltetrazolium bromide (MTT) assay. Following a 48 h EV treatment, 50 μls of a 5 mg/ml MTT solution was added to each well for 2 h. Cells were then dissolved in 100 μl of DMSO and absorbance was measured at 570 nm. Endothelial cell counts were measured following 48 h EV treatment using a hemocytometer (Hausser Scientific, Horsham, PA, USA).

### Endothelial tube formation and migration

A tube formation assay was performed in reduced growth factor Matrigel (Corning, Corning, NY, USA) coated 96-well plates. Plates and pipette tips were chilled at – 20 °C and the Matrigel was thawed in ice at 4 °C overnight. Each well was coated with 50 μl of Matrigel and incubated for 30 min at 37 °C. During incubation, HUVECs were trypsinized and counted in serum free Dulbecco’s Modified Eagle Medium (DMEM; Sigma-Aldrich). 15,000 cells were seeded per well and non-control cells were treated with OXI- or GLY-EVs. Cells were imaged at 25 × magnification on a Leica DMi6000 microscope following a 6 h incubation. Images were analyzed using the angiogenesis analyzer tool on ImageJ (National Institutes of Health, Bethesda, MD, USA). Total tube length and number of tubes per field of view were measured.

Migration was analyzed via a scratch assay. HUVECs were grown to 100% confluence on a 24-well plate. A pipette tip (1000 μl) was used to scratch down the middle of each well, detaching the cells in that area. Detached cells were removed and the media was replaced with EBM supplemented with 2% EV-depleted FBS. Non-control cells were treated with OXI- or GLY EVs. Following an 8 h incubation, all cells were fixed in 4% PFA. Cells were imaged twice at 25 × magnification, immediately following the scratch, but prior to EV treatment, and after an 8 h incubation. Each post-incubation image was compared to its pre-treatment image for analysis. The number of migrated cells were counted using the multi-point counter tool on ImageJ.

### Quantitative real-time PCR

Total RNA was extracted from EVs and from endothelial cell samples following a 48 h EV incubation using Trizol reagent (Thermo Fisher) according to the manufacturer’s instructions. RNA concentration was determined by Nano-Drop (Thermo Fisher). RNA concentration of each sample was normalized and first-strand cDNA was generated with MMLV Reverse Transcriptase and random hexamer primers (Invitrogen). Real-time PCR was performed using primers and SYBR green based chemistry on a CFX connect Real-Time PCR system (Bio-Rad, Hercules, CA, USA). Primer sequences were obtained from the Harvard Primer Bank. The 2^−ΔΔCt^ relative quantification method was used to calculate gene expression. Data were normalized to GAPDH.

### Immunoblotting

Total protein was isolated from EV and HUVECs, following a 48 h EV incubation, using radioimmunoprecipitation assay (RIPA) buffer (50 mM Tris–HCl (pH 7.4), 150 mM NaCl, 2 mM EDTA, 0.1% SDS, 0.1% Triton X-100, and 0.5% Deoxycholate) with protease inhibitor (Thermo Fisher) and phosphatase inhibitors (50 mM NaF and 0.2 mM Na3VO4). Total protein concentration was determined via BCA assay. Protein concentrations of each sample were equalized to each other and 20–30 μg were loaded into SDS–polyacrylamide gels. Following electrophoresis, protein was transferred to polyvinylidene difluoride (PVDF) membranes. Membranes were incubated in blocking buffer, 5% non-fat milk dissolved in TBST (25 mM Tris (pH 7.2), 150 mM NaCl, and 0.1% Tween 20) for 1 h at room temperature. Membranes were incubated with primary antibodies (ALIX, cat#2171; CD63, cat#15363; Clathrin, cat#4796; Cytochrome c, cat#136F3; eNOS, cat#9572; p-eNOS, cat#9571; Akt, cat#9272; p-Akt, cat#9271; α-tubulin, cat#32-2500) (Cell Signaling Technology, ThermoFisher Scientific, and Santa Cruz Biotechnology) in 5% non-fat milk dissolved in TBST overnight at 4 °C. Membranes were washed in TBST and incubated in secondary antibody, anti-rabbit or anti-mouse immunoglobulin G-horseradish peroxidase (Cell Signaling Technology, Danvers, MA, USA) for 1 h. Images were obtained by chemiluminescence using ChemiDoc Touch Imaging System (BioRad). Densitometric analysis was performed (Image Lab software; Biorad) and protein concentration was normalized to tubulin.

### Statistical analysis

Analyses were conducted via either student’s t-test, one-way, or two-way ANOVA. Tukey’s or Fisher’s LSD post-hoc comparisons were run when appropriate. All analyses were done using GraphPad Prism (Version 9.2; GraphPad Software Inc., San Diego, CA, USA). *P* < 0.05 was considered statistically significant for all statistical sets. Data are presented as the mean ± SD (Supplementary Information).

### Supplementary Information

Supplementary Information.

## Data Availability

Sequencing data sets associated with this study are available on the NCBI GEO database (accession #GSE216121). Raw data can be made available from the corresponding author upon reasonable request.
