# Supplementary material for: Angiogenic potential of skeletal muscle derived extracellular vesicles differs between oxidative and glycolytic muscle tissue in mice
Source: Sci Rep. 2023 Nov 2;13:18943. doi: 10.1038/s41598-023-45787-9 (PMC10622454; doi:10.1038/s41598-023-45787-9)

Extracellular vesicle EV Western blots:

SOD3: Loaded as OXI-EV; GLY-EV


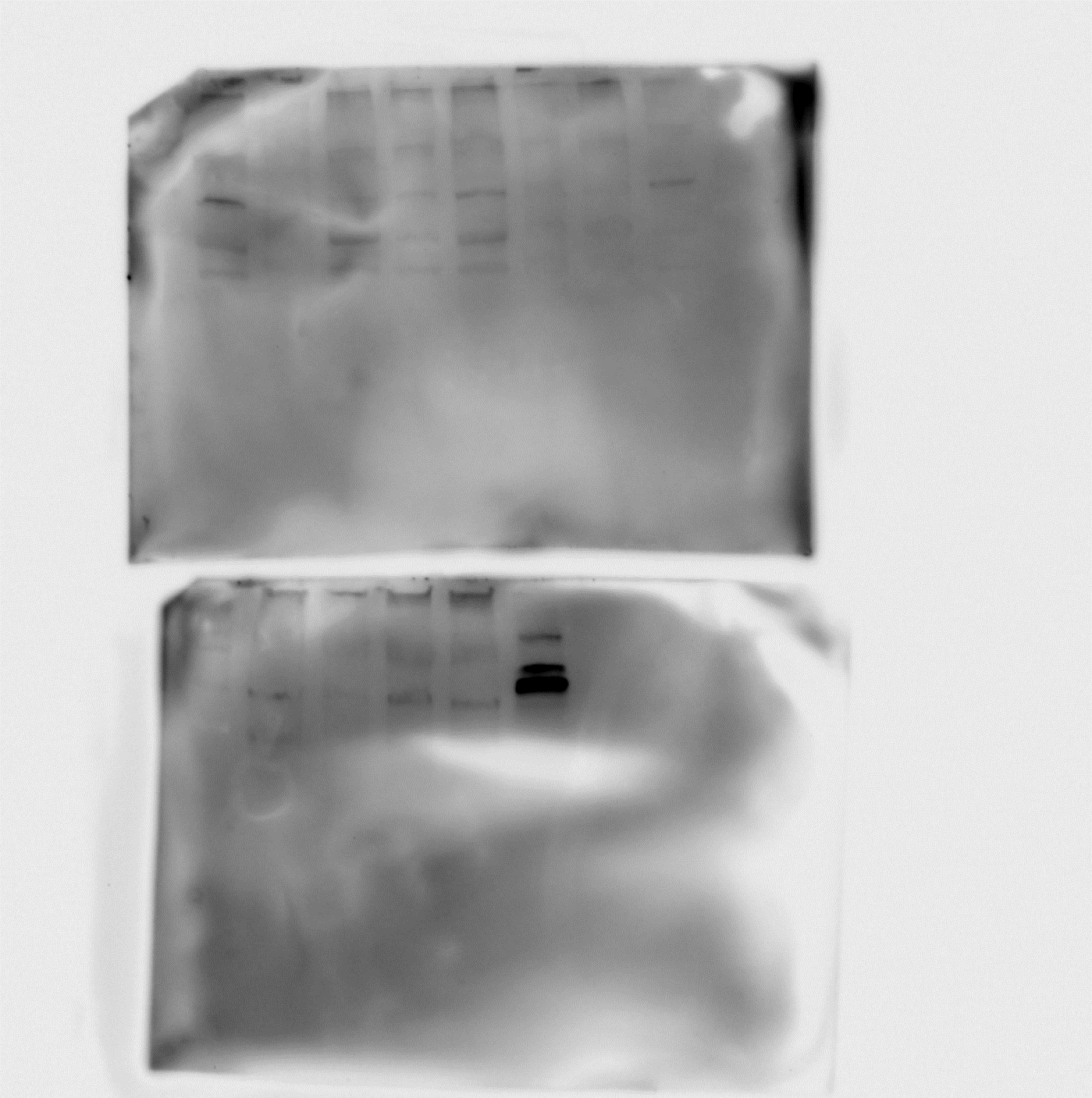


37 kDa marker; SOD3 bands at 34 kDa

37 kDa marker; SOD3 bands at 34 kDa

Gels were loaded in following order:

CON; GLY EV; OXI EV

EV VEGF: Loaded as OXI-EV; GLY-EV


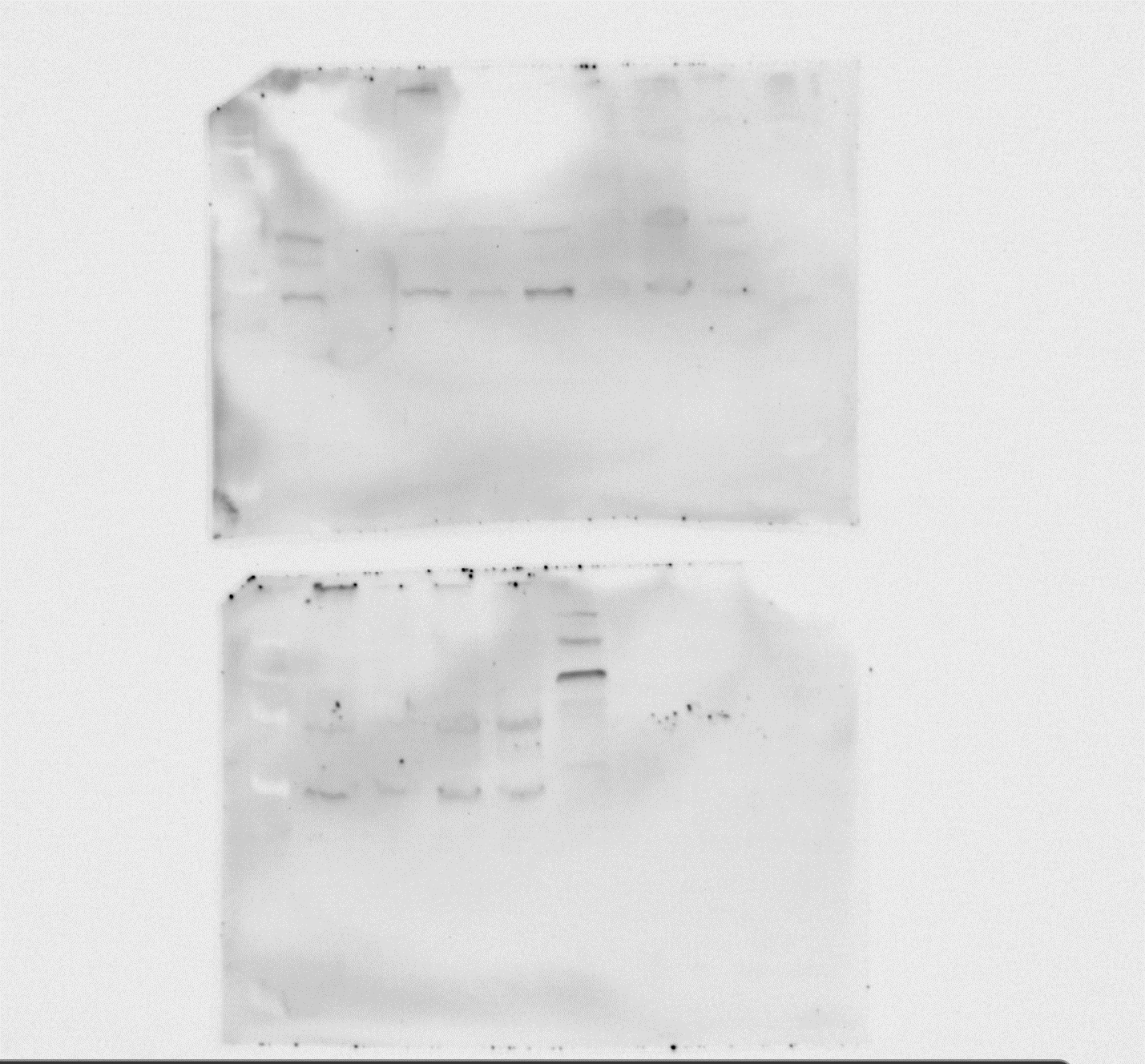


25 kDa marker; VEGFA band right below 25 kDa

25 kDa marker; VEGFA band right below 25 kDa

EV total protein blot: Loaded as OXI-EV; GLY-EV. Markers are first lane on each blot


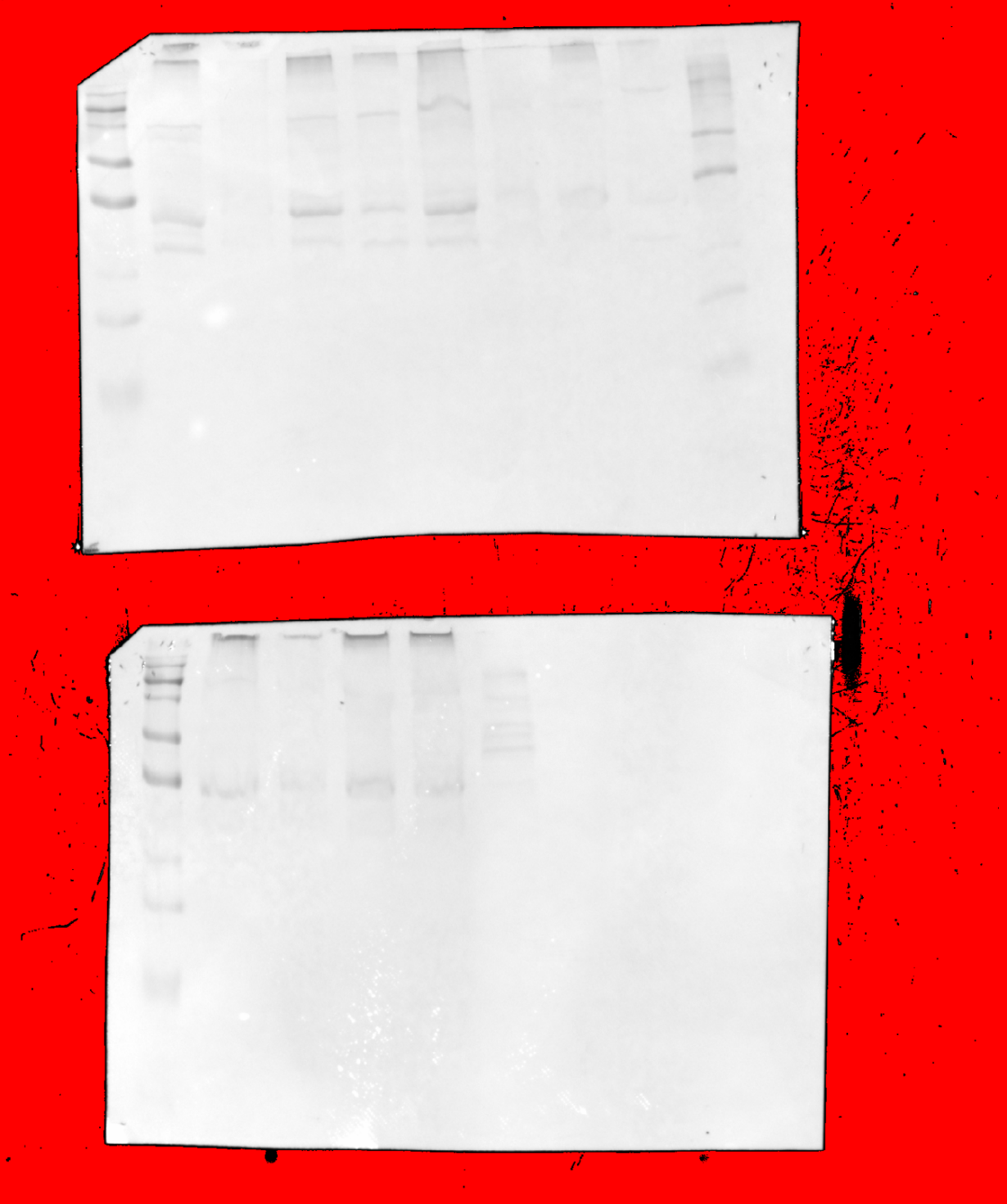


EV TSG101 (pooled samples; not meant for quantification; ran to identify presence of the protein in the EV samples):


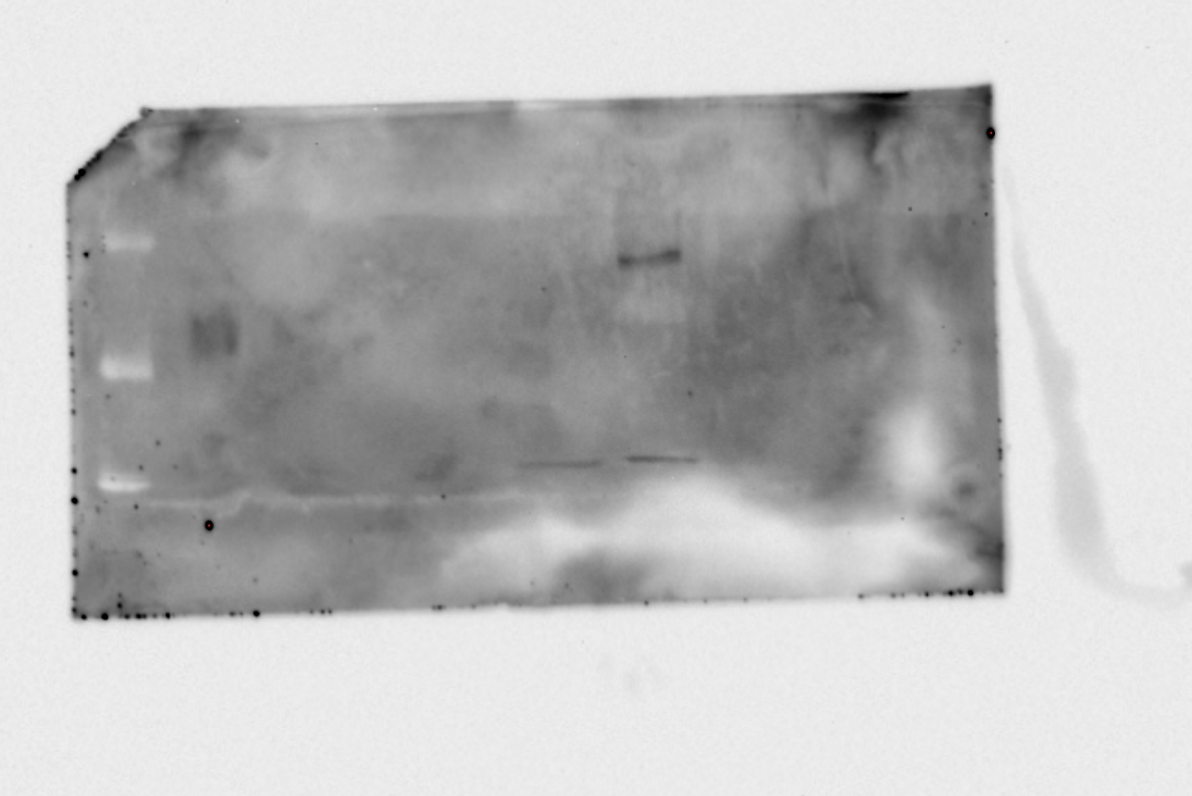


Loaded as: OXI-EV, GLY-EV

EV CD63 (pooled samples; not meant for quantification; ran to identify presence of the protein in the EV samples):


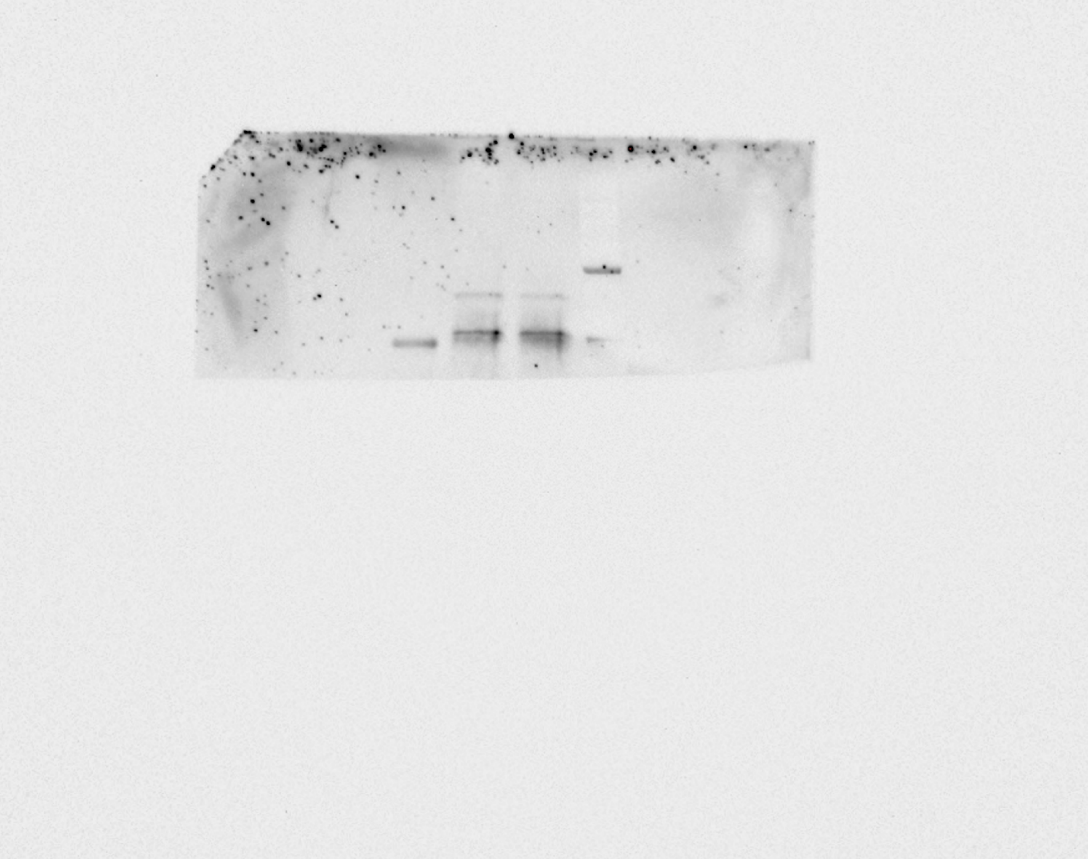


Loaded in following order:

OXI-EV, GLY-EV

EV cytochrome 63 (pooled samples; not meant for quantification;ran as a negative control for absence of the protein in the EV samples): Loaded as Control-EV, OXI-EV, GLY-EV, Myotube Control 1, Myotube Control 2


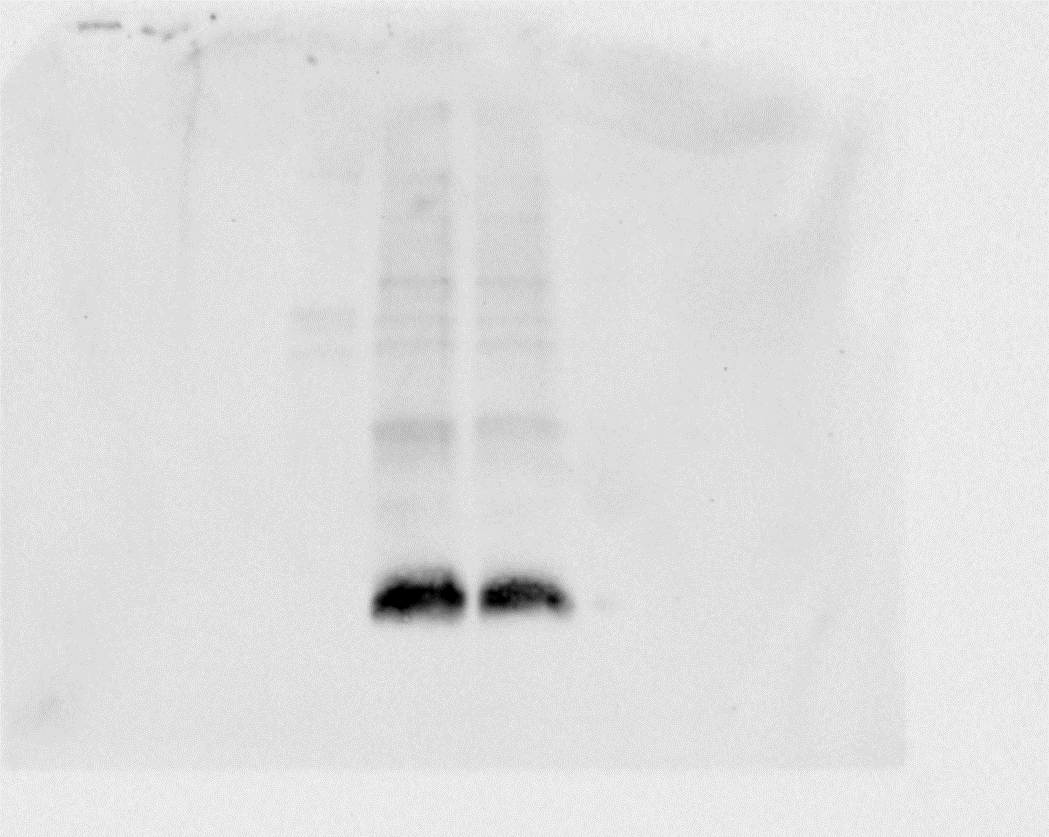


EVs

Myotube controls

NOTE TO EDITORS/REVIEWERS: The order that the western blot samples were loaded into the gel (Control, OXI-EV (or OXI-EV treated ECs), GLY-EV (or GLY-EV treated ECs) are consistent for all blots except the t- and p-Akt western blots. These were run in the order of CON, GLY EV, OXI-EV. In the manuscript the representative images of Figure 3 were rearranged so that the images are in a consistent order. This has been done to aid the reader but if the Reviewers/Editors would like the authors to alter how we displayed the representative images we are happy to comply.

p-eNOS


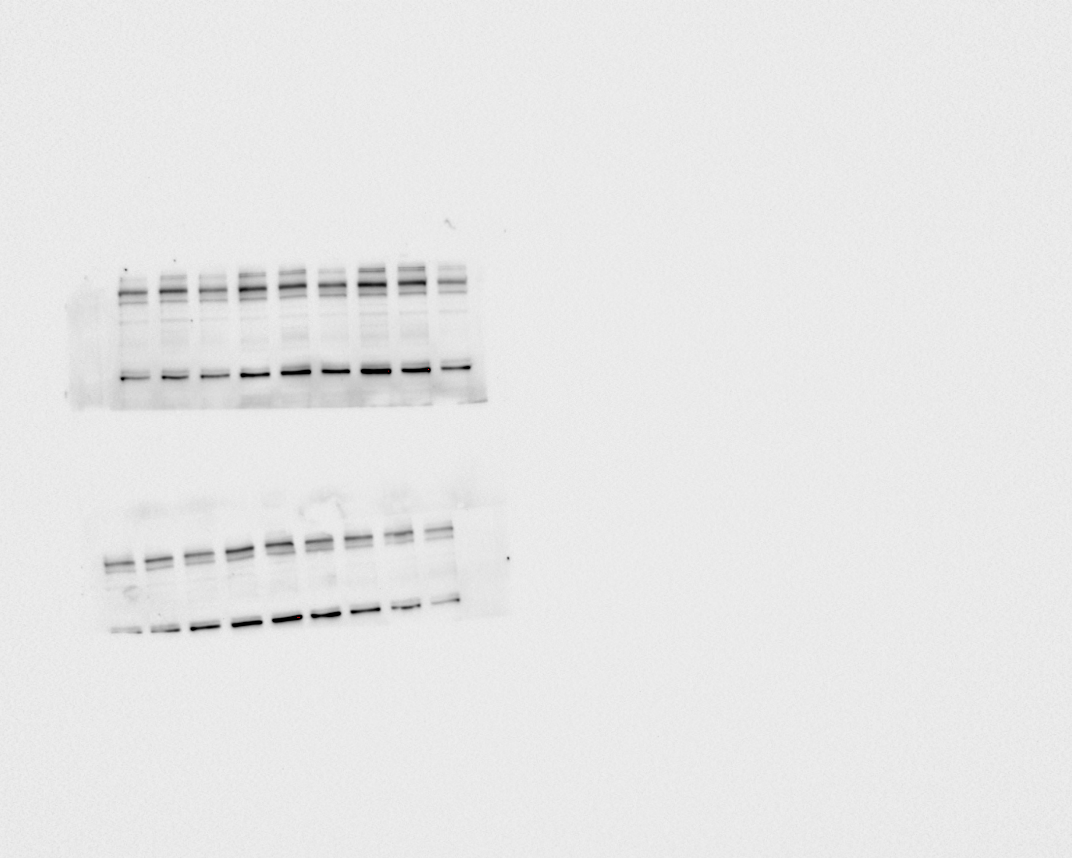


Gels were loaded in following order:

CON-OXI EV-GLY EV

140 kDa

140 kDa

t-eNOS


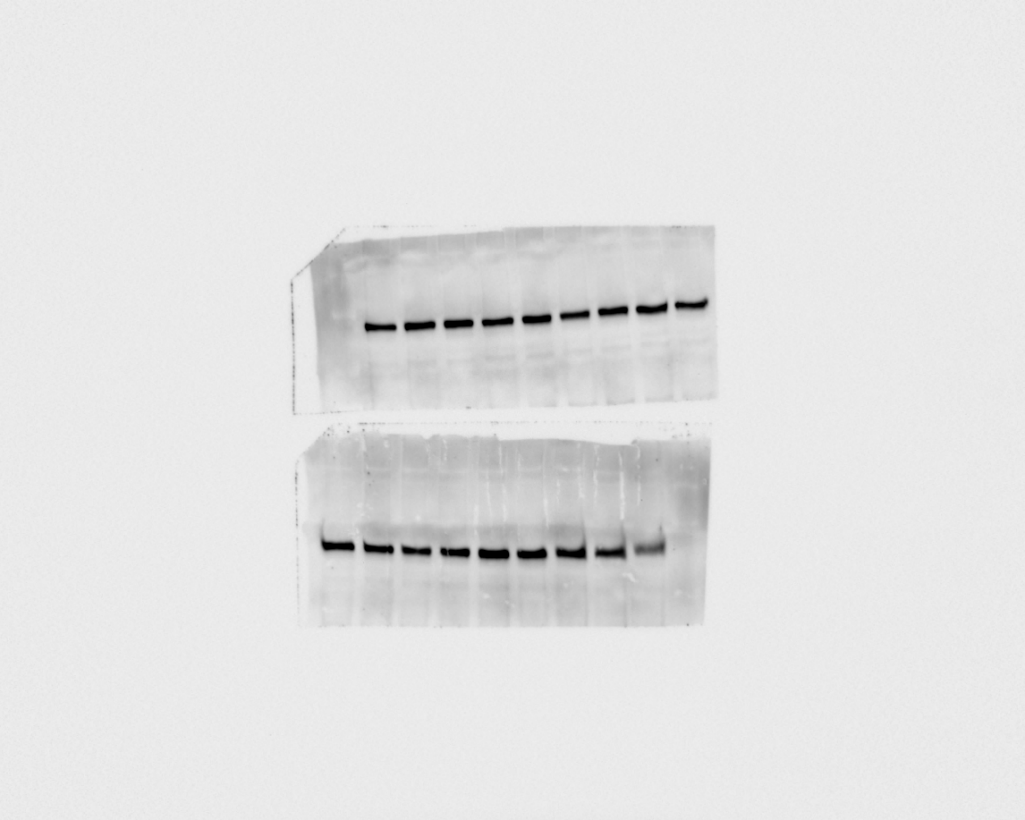


140 kDa

140 kDa

Gels were loaded in following order:

CON-OXI EV-GLY EV

Tubulin:

Gels were loaded in following order:

CON-OXI EV-GLY EV


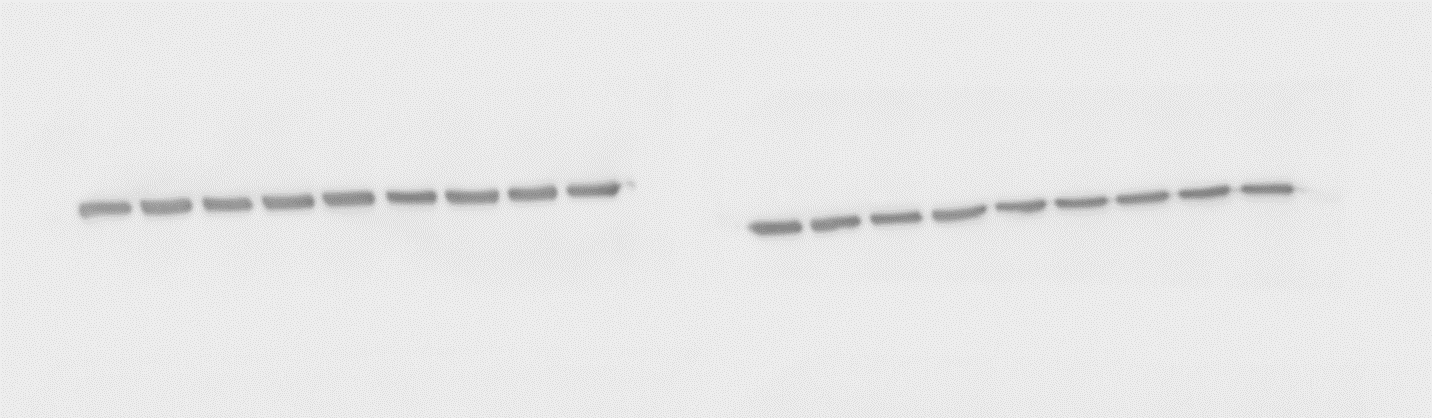


p-Akt:


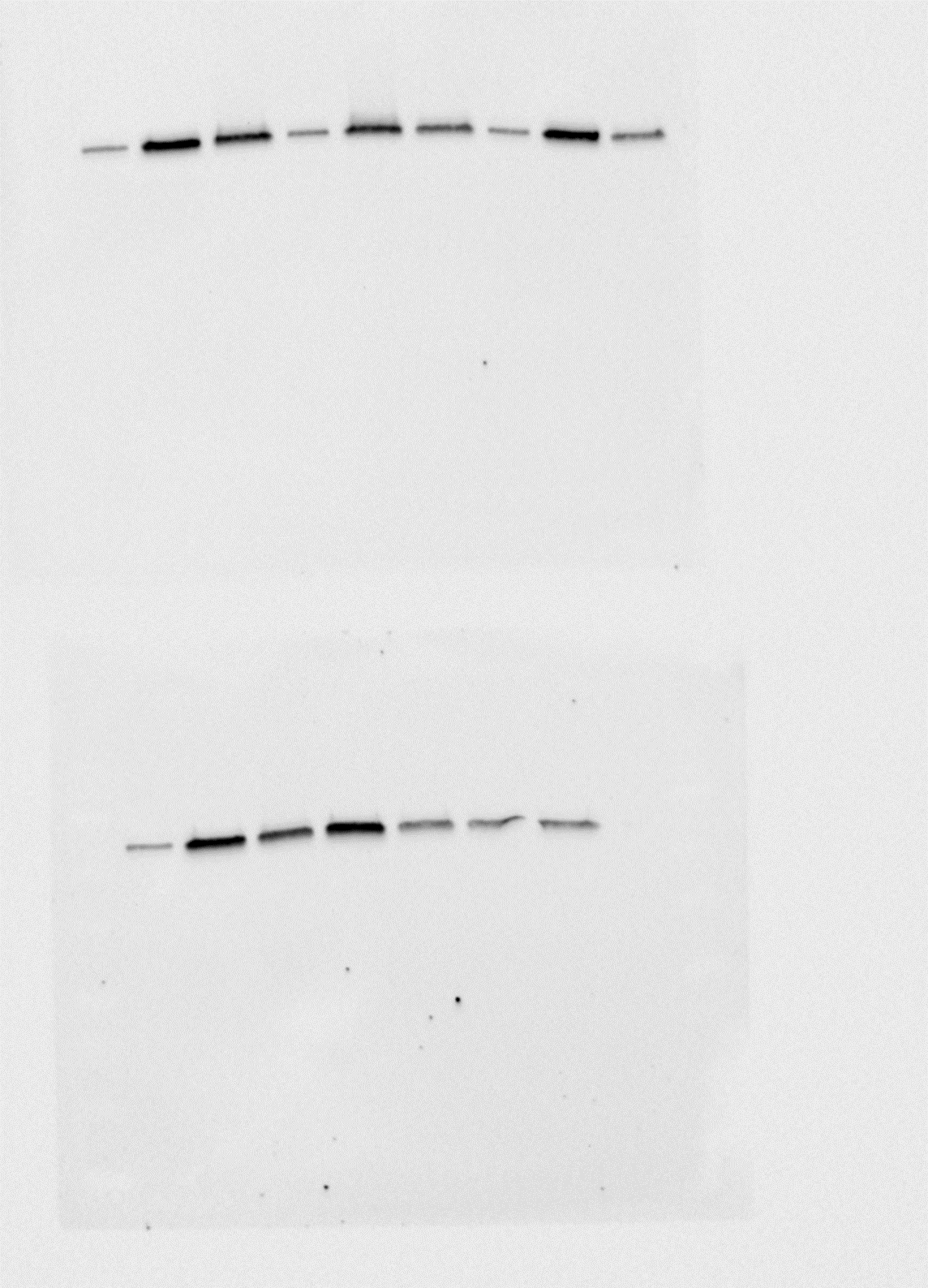


Gels were loaded in following order:

CON; GLY EV; OXI EV

t-Akt:


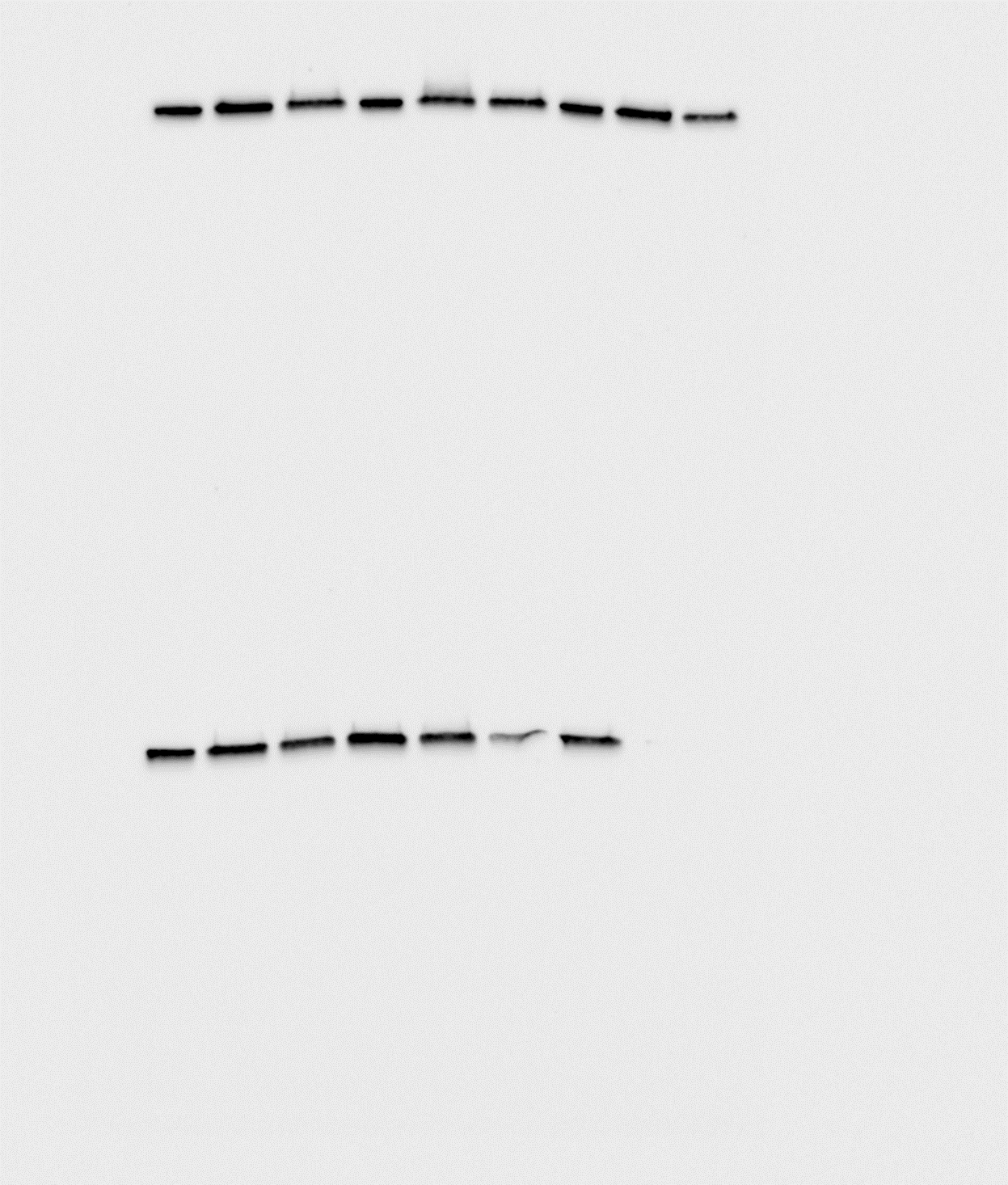

Supplement: Supplementary file 1 — Supplementary Information. [file 41598_2023_45787_MOESM1_ESM.docx]
